# A CFH peptide-decorated liposomal oxymatrine inactivates cancer-associated fibroblasts of hepatocellular carcinoma through epithelial–mesenchymal transition reversion

**DOI:** 10.1186/s12951-022-01311-1

**Published:** 2022-03-05

**Authors:** Jian Guo, Huating Zeng, Xinmeng Shi, Tao Han, Yimin Liu, Yuping Liu, Congyan Liu, Ding Qu, Yan Chen

**Affiliations:** 1 Affiliated Hospital of Integrated Traditional Chinese and Western Medicine, Nanjing University of Chinese Medicine, Nanjing, 210028 China; 2 Jiangsu Province Academy of Traditional Chinese Medicine, 100 Shizi Road, Nanjing, 210028 China; 3 Anhui Province Key Laboratory of Pharmaceutical Preparation Technology and Application, Engineering Technology Research Center of Modern Pharmaceutical Preparation, College of Pharmacy, Anhui University of Chinese Medicine, 230012 Hefei, China

**Keywords:** Liposomal oxymatrine, Cancer-associated fibroblasts, CFH peptide, Epithelial–mesenchymal transition, Tumor microenvironment, Hepatocellular carcinoma

## Abstract

Cancer-associated fibroblasts (CAFs) deteriorate tumor microenvironment (TME) and hinder intra-tumoral drug delivery. Direct depleting CAFs exists unpredictable risks of tumor metastasis. Epithelial–mesenchymal transition (EMT) is a critical process of CAFs converted from hepatic stellate cells during hepatocellular tumorigenesis; however, until now the feasibility of reversing EMT to battle hepatocellular carcinoma has not been comprehensively explored. In this study, we report a CFH peptide (CFHKHKSPALSPVGGG)-decorated liposomal oxymatrine (CFH/OM-L) with a high affinity to Tenascin-C for targeted inactivating CAFs through reversing EMT, which is verified by the upregulation of E-cadherin and downregulation of vimentin, N-cadherin, and snail protein in vivo and in vitro. After the combination with icaritin-loaded lipid complex, CFH/OM-L obviously boosts the comprehensive anticancer efficacy in both 3D tumor spheroids and stromal-rich tumor xenograft nude mouse models. The combinational therapy not only effectively reversed the in vivo EMT process but also significantly lowered the collagen, creating favorable conditions for deep penetration of nanoparticles. More importantly, CFH/OM-L does not kill but inactivates CAFs, resulting in not only a low risk of tumor metastasis but also a reprogramming TME, such as M1 tumor-associated macrophages polarization and natural killer cells activation. Such strategy paves a moderate way to remold TME without depleting CAFs and provides a powerful tool to design strategies of combinational hepatocellular carcinoma therapy.

**Graphical Abstract:**

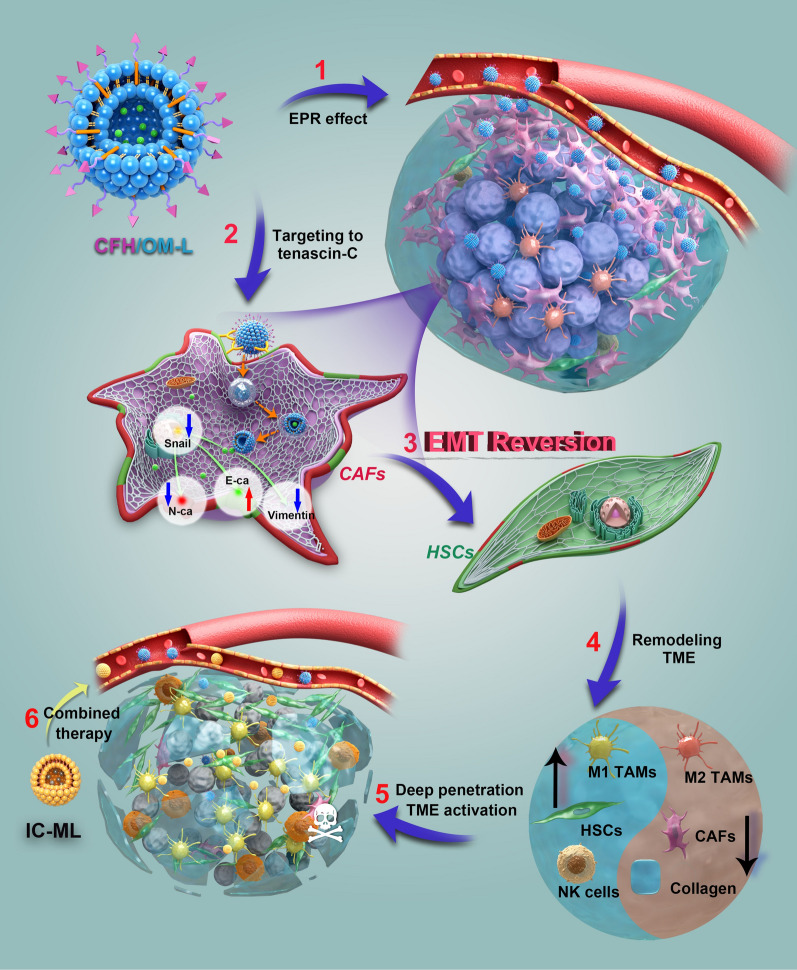

**Supplementary Information:**

The online version contains supplementary material available at 10.1186/s12951-022-01311-1.

## Background

Cancer-associated fibroblasts (CAFs) increasingly attract much attention in the treatments of various desmoplastic tumors treatment [1–5]. As the most population of stromal cells, CAFs are responsible for the tumor microenvironment (TME) with plenty of collagen, fibronectin and proteoglycans, which hinder the deep penetration of nanoparticles into the inner of the tumors. In addition, CAFs also secrete various types of cytokines, such as transforming growth factor-β (TGF-β), hepatocyte growth factor, C-X-C motif chemokine ligand 12 (CXCL12), and platelet-derived growth factor, which are one of the main culprits for the pro-tumoral malignant environment. Furthermore, CAFs usually distribute between the tumor and vascular epithelial cells, that could compete with tumor cells to eat the nanoparticles leaking from the blood vessels [6]. According to the current mainstream consensus, CAFs play a central role in the crosstalk between various immune signals in the tumor, and also have been considered as the most promising breakthrough to reshape the TME [7].

Previous reports have validated that depleting CAFs was able to improve the anticancer efficacy in the treatments of prostate, breast, and pancreatic cancer; however, the researchers also alert the consequential risk of tumor migration [8, 9]. In addition, further TME immunosuppression has to be faced up because abnormally-increased CD4^+^Foxp3^+^ Tregs have been observed in myofibroblast-depletion tumors [9]. Regulating the behavior of CAFs in the TME, instead of direct killing, is becoming a promising way to improve the malignant TME. Recently, Nie and coworkers inactivated CAFs biofunctions via silencing CXCL12, such a non-CAFs depletion strategy effectively enhanced the anticancer efficacy via remolding the TME [10].

During hepatocellular tumorigenesis, epithelial–mesenchymal transition (EMT) is the critical step that regulates the transformation of hepatic stellate cells to CAFs [11]. However, until now we still know little about the feasibility of EMT reversion for CAFs inactivation and TME activation in hepatocellular carcinoma therapy. As reported previously, the intervention of EMT achieved a few positive responses in the treatment of breast cancer [12, 13]. In view of this, we hypothesize that interfering with the process of EMT might block the production of CAFs and thereby counteract its role of bridge of immune crosstalk in TME. Unfortunately, few small-molecule compounds have been demonstrated with a definite reversion toward EMT in hepatocellular carcinoma so far.

Oxymatrine (OM), a quinolizidine alkaloid extracted from the dry roots of *Sophora flavescens*, is capable of balancing the synthesis-degradation of extracellular matrix and controlling the collagen deposition in liver. Notably, OM can also reduce the secretion of TGF-β1 through down-regulating the high mobility group box-1, which deactivated TGF-β1-mediated activation of hepatic stellate cells [14]. Combined with these information, we believe that OM is a promising compound that could be used for reversing EMT and inactivating CAFs in the TME of hepatocellular carcinoma. Based on the preliminary study, we found that the non-selective biodistribution and undesired physicochemical properties of OM are limiting the application of EMT reversion [15, 16]. A suitable drug delivery is highly desired for OM.

**Scheme 1:**
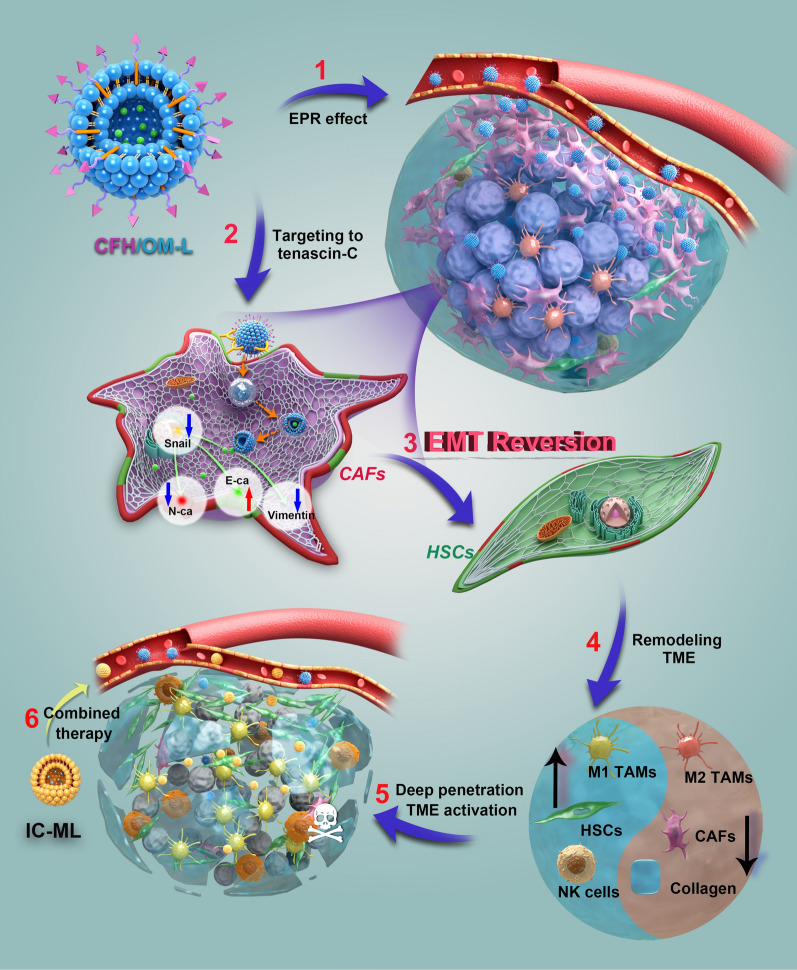
Schematic diagram of CFH/OM-L reversing EMT process of CAFs activation derived from hepatic stellate cells (HSCs) and consequent boosting hepatocellular carcinoma therapy with IC-ML. After accumulation at hepatocellular carcinoma sites, CFH/OM-L actively enters into TGF-β1-activated CAFs via high affinity between CFH peptide and tenascin-C. CFH/OM-L can educate activated CAFs into quiescent HSCs through EMT reversion, which is proofed by the upregulation of E-cadherin and the downregulation of vimentin, N-cadherin, and snail protein. The reduction of CAFs leads to TME reprogramming, such as collagen reduction, M1 tumor-associated macrophages (TAMs) polarization, NK cells activation, and so on. The CFH/OM-induced TME activation not only damages tumorigenesis but also assists IC-ML to penetrate deeply, thereby enhancing the comprehensive anticancer efficacy

Tenascin-C, overexpressing in CAFs of stroma-rich solid tumors [17, 18], has a high affinity to the fragment of FH peptide, presenting a potential CAF-targeted ligand for drug delivery [19, 20]. Herein, we design a new cysteine-end FH peptide (CFH) (CFHKHKSPALSPVGGG) and decorate on the surface of OM-loaded liposomes (CFH/OM-L), aiming for CAFs-targeted delivery and EMT reversion. As depicted in Scheme 1, CFH/OM-L has a high affinity with tenascin-C overexpressing on the surface of CAFs, reverses the EMT process, lowers the collagen deposition, and reduces CAFs abundance, thereupon then creating a favorable environment for deep penetration of nanoparticles and activating the TME for boosted anticancer efficacy. Furthermore, the lipid complex co-loading icaritin and coix seed oil (IC-ML), a reported anti-angiogenesis nanoparticle is combined with CFH/OM-L to validate the rationality of our design [21]. Benefiting from the improvement on intra-tumoral drug delivery, as well as the reshaped TME, IC-ML penetrates deeply inside the tumor and presents the more powerful anticancer ability against hepatocellular carcinoma. Overall, the CAFs-targeted delivery of OM performed by CFH/OM-L exhibits a safe and effective approach to improve the drug delivery and TME, making a valuable reference for the stromal-rich tumor therapies.

## Results and discussion

### Synthesis of DSPE-PEG_2000_-CFH

The synthetic route of DSPE-PEG_2000_-CFH was shown in Additional file 1: Fig. S1. The CFH peptide was conjugated with DSPE-PEG_2000_-MAL via the Michael addition between the thiol segments and the double bonds of maleic groups [22]. As shown in Additional file 1: Fig. S2, The S-H stretching vibration of CFH peptide was observed in a range of 2500–2600 cm^−1^. Notably after linked with DSPE-PEG_2000_, the signal of the thiol groups was disappeared. In addition, the N–H stretching vibration at ~ 3360.5 cm^−1^ and C=O stretching vibration at ~ 1712.1 cm^−1^ of DSPE-PEG_2000_-CFH were both significantly stronger than the bare lipid. As for the ^1^H-NMR characterization, the peaks at ~ 6.77 ppm of DSPE-PEG_2000_-MAL ascribed as the MAL groups disappeared after conjugation with CFH peptide (Additional file 1: Fig. S3), suggesting a complete reaction between the thiol and MAL groups. According to the MALDI-TOF MS spectrum, the molecular weight of DSPE-PEG_2000_-CFH was measured as ~ 4100 (Additional file 1: Fig. S4), which was in accordance with the theoretical molecular weight sum of CFH peptide and DSPE-PEG_2000_-MAL. All the obtained results suggest a successful synthesis of DSPE-PEG_2000_-CFH.

### Preparation and characterization of CFH/OM-L

**Fig. 1:**
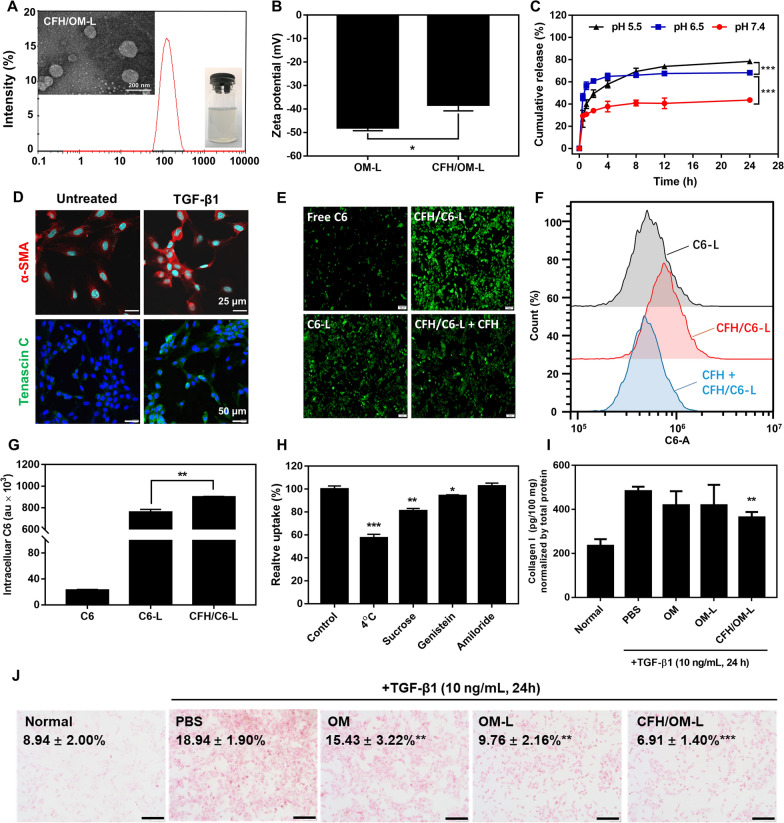
Liposome characterizations, cellular uptake and inhibition of collagen expression in CAFs. **A** Appearance, particle size, and TEM image of CFH/OM-L. **B **Zeta potential of OM-L and CFH/OM-L. Data are represented as mean ± SD, n = 3, *P < 0.05. **C** Accumulative release profile of OM from CFH/OM-L in PBS with different values. Data are represented as mean ± SD, n = 3, ***P < 0.001. **D** Immunofluorescence images of α-SMA and Tenascin C in LX-2 and TGF-β1-activated LX-2 cells. **E** Fluorescence images of CAFs uptake after treatments with various formulations. Scale bar: 100 μm. **F** CAFs uptake of C6-L, CFH/C6-L, and CFH/C6-L + CFH peptide detected by flow cytometry. (**G**) Intracellular fluorescence intensity measured by flow cytometry. Data are represented as mean ± SD, n = 3. **P < 0.01. **H** Cellular uptake mechanism studied by specific internalization inhibitors. Data are represented as mean ± SD, n = 3. Compared with the control group, *P < 0.05, **P < 0.01, ***P < 0.001. **I** The expression of Collagen I in various formulation groups measured by ELISA assay. Data are represented as mean ± SD, n = 3. Compared with the model group, **P < 0.01. **J** Picrosirius red staining of total collagen in various formulation groups. Scale bar: 200 μm. The positive staining cells were quantified in 3 randomly selected fields per section. Data are represented as mean ± SD, n = 3. Compared with the PBS group, **P < 0.01

CFH/OM-L was prepared by a pH-gradient method as reported previously [16]. As shown in Table 1; Fig. 1A, the average particle size and EE of CFH/OM-L were 125.30 ± 0.96 nm and 87.12 ± 0.78%, respectively. The morphology of CFH/OM-L displayed as spherical particles around 120 nm with a narrow dispersion. The zeta potential of CFH/OM-L was slightly higher than that of OM-L (Fig. 1B), which was probably due to the shielding of CFH peptide on the outer layer. The particle size and zeta potential of CFH/OM-L did not display significant changes regardless of the incubation with mice plasma for 24 h or the treatment with phosphate buffered saline (PBS) for 40 days (Additional file 1: Fig. S5A, B). As shown in Additional file 1: Fig. S5C, both of CFH/OM-L and OM-L were capable of sustainedly releasing OM in PBS of pH 7.4. With the decrease of pH, the OM was released much faster from CFH/OM-L than that in pH 7.4. The 24 h-cumulative release of OM from CFH/OM-L was 43.58 ± 1.25% and 78.49 ± 1.33% in pH 7.4 and 5.5, respectively (Fig. 1C). It suggests that CFH/OM-L may slowly release OM in normal tissues, and unload cargo faster after entering tumor tissues and being internalized by targeted cells. Besides, the pharmaceutical characterizations of IC-ML were also studied as exhibited in Additional file 1: Fig. S6, which was similar to our previous report [21].

**Table 1:** Pharmaceutical characterizations of OM-L and CFH/OM-L

Formulations	Size (nm)	PDI	Zeta potential (mV)	EE (%)
OM-L	116.90 ± 1.10	0.09 ± 0.01	− 48.00 ± 1.21	82.25 ± 0.93
CFH/OM-L	125.30 ± 0.96	0.08 ± 0.02	− 38.30 ± 2.47	87.12 ± 0.78

Data are represented as mean ± SD, n = 6

### CAFs cellular uptake of CFH/OM-L

The in vitro CAFs model of hepatocellular carcinoma was built as reported previously [23]. As shown in Fig. 1D, the expression of α-SMA in LX-2 cells significantly increased after the treatment with TGF-β1. Likewise, the fluorescence signal of Tenascin C in CAFs model was obviously stronger than that of the untreated group, which was in accordance with the previous report [23]. Tenascin C is one of the most characteristic makers expressed in CAFs, it has been considered as a potential ligand for targeted drug delivery to the CAFs in the tumor microenvironment. The peptide sequence of CFH with a high affinity to tenascin C is designed through phage display peptide library selection. The binding affinity of CFH peptide to tenascin C reached 4.58 ± 1.4 µM detected by Surface Plasmon Resonance, which is one of the most specific peptides for CAFs in published papers [19]. As for the internalization studies, the intracellular fluorescence of CFH/C6-L-treated cells was significantly higher than that of C6-L group, in addition, such an enhancement could be attenuated by the competitive inhibition with free CFH peptide (Fig. 1E–G). The above-mentioned results indicate that CFH/C6-L enters into the CAFs in the Tenascin C-mediated pathway. As presented in Fig. 1H, the internalization of CFH/C6-L was suppressed in the presence of sucrose and genistein. Besides, low temperature also hinders the cellular uptake of CFH/C6-L. These results suggest that CAFs cellular uptake of CFH/OM-L is probably associated with the Tenascin C-, clathrin- and caveolin-mediated pathways with the involvement of energy.

### Expression of collagen in CAFs in vitro

CAFs are the most abundant stromal cells in the TME, which produce excessive collagen deposition to hinder intra-tumoral deep penetration of nanoparticles [24]. Herein, we investigated whether CFH/OM-L could inhibit the collagen secretion of CAFs in vitro. As shown in Fig. 1I, the collagen I secretion of CAFs was increased by the activation of TGF-β1. However, when CAFs treated with CFH/OM-L, the expression of collagen I was greatly reduced compared with the model group (*P* < 0.01). Furthermore, the picrosirius red staining method was also employed to evaluate the expression of collagen. The images presented that the treatment with OM-L and CFH/OM-L can both reduce the level of collagen (Fig. 1J). The aforementioned results indicate that CFH/OM-L effectively inhibits the collagen secretion of CAFs in vitro, which is beneficial to reduce the delivery resistance of nanomedicines in tumor stroma.

### EMT reversion in vitro

**Fig. 2:**
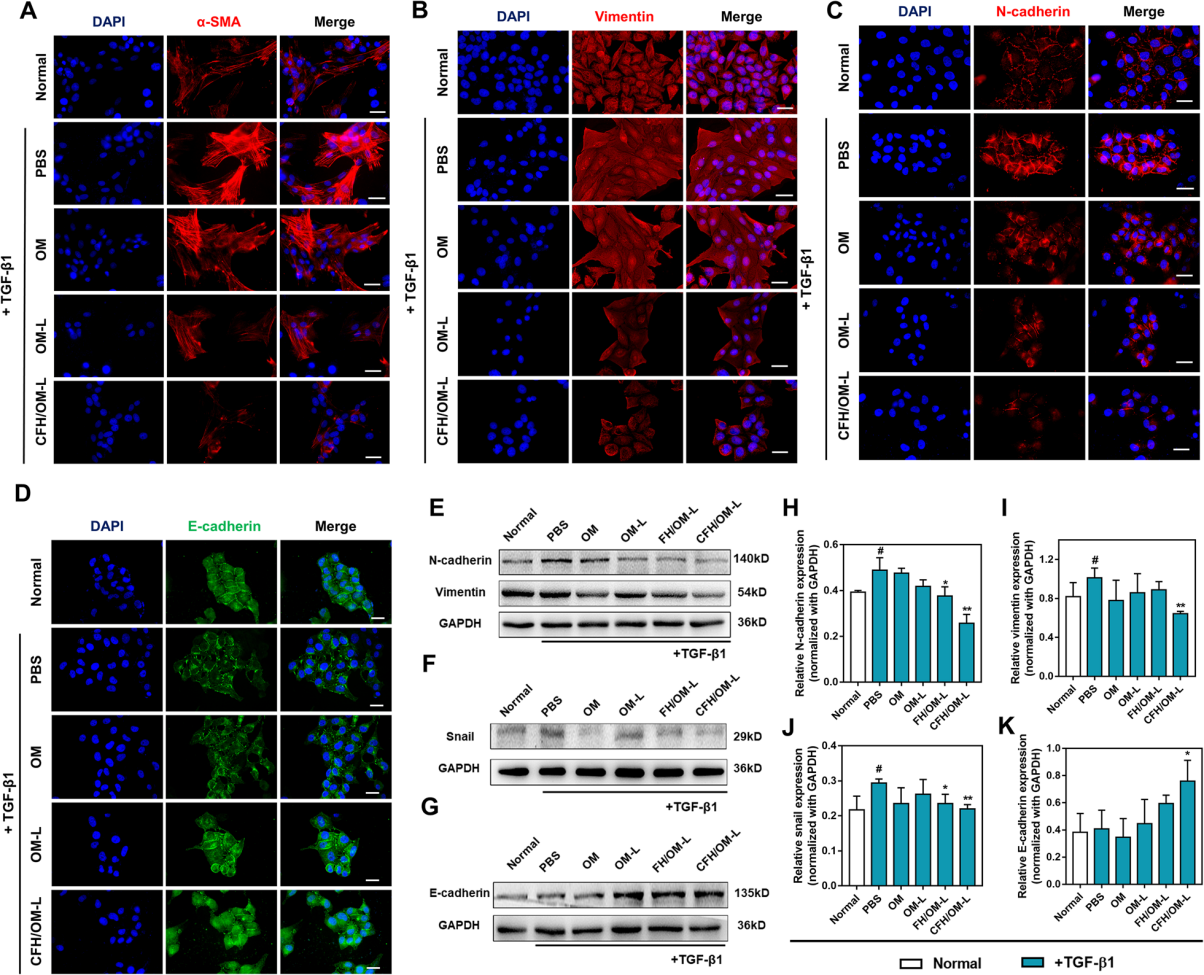
CFH/OM-L reverses CAFs activation and EMT in vitro. Immunofluorescence staining of **A** α-SMA, **B** vimentin, **C** N-cadherin and **D** E-cadherin after TGF-β1-activated LX-2 cells were treated with various formulations. Scale bar: 20 μm. Representative images of western blot depicting bands for **E** N-cadherin, vimentin, **F** snail and **G** E-cadherin in various formulation groups. Quantification of **H** N-cadherin, **I** vimentin, **J** snail and **K** E-cadherin expression after treatments with different formulations. Data are represented as mean ± SD, n = 3. Compared with the normal group, ^#^*P* < 0.05; compared with the PBS group, *P < 0.05, **P < 0.01

EMT is considered as one of the most significant events in the transformation of hepatic stellate cells into CAFs, leading to an obstacle of intracellular drug delivery and promoting tumor metastasis. Although previous studies observed the reduction of CAFs in several OM-based anticancer treatments, including breast cancer, colon cancer and non-small lung cancer [25–27], its feasibility of regulation of EMT toward CAFs in hepatocellular carcinoma is still uncertain until now. To investigate whether CFH/OM-L inhibits the activation of CAFs through regulating EMT, the characteristic proteins of CAFs and EMT were observed by immunofluorescence staining. As shown in Fig. 2A–C, the expression of α-SMA, vimentin, N-cadherin of TGF-β1-activated LX-2 cells were significantly decreased after treatments with CFH/OM-L and OM-L. Notably, free OM hardly worked on EMT process, which was ascribed to its low internalization. The expression of characteristic proteins of EMT were also investigated by western blot method. As shown in Fig. 2E, F, H–J, CFH/OM-L can remarkably downregulate the expression of N-cadherin, vimentin and snail compared with the PBS-treated group. Furthermore, CFH/OM-L was capable of increasing the level of E-cadherin (Fig. 2D), which was significantly higher than the PBS-treated group (Fig. 2G, K). The above-mentioned effects of CFH/OM-L are almost as powerful as that of FH/OM-L. In view of that FH peptide is a known ligand of affinity with Tenascin C [23], the advantages of EMT reversion of CFH/OM-L over OM-L are probably related to the improved internalization.

### In vivo distribution studies

**Fig. 3:**
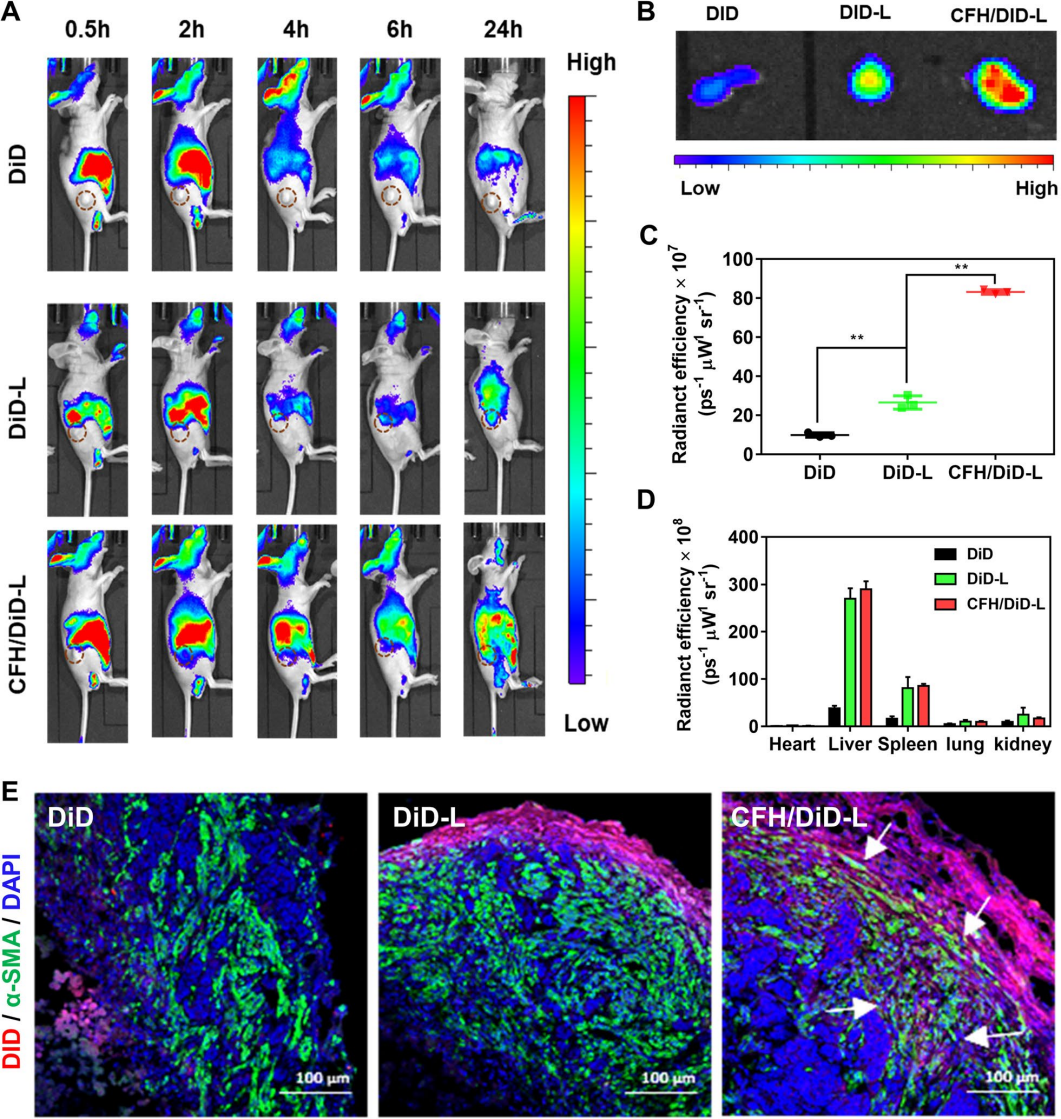
Distribution and penetration of DiD formulations in vivo. **A** Biodistribution of DiD signal in stromal-rich xenograft-bearing nude mice treated with free DiD, DiD-L, CFH/DiD-L at predetermined intervals. **B** The fluorescence images of ex vivo tumors in various formulation groups at 24 h post-administration. The fluorescence intensity of ex vivo (**C**) tumors and **D** normal tissues of xenograft-bearing nude mice treated with various formulations at 24 h post-administration. Data are represented as mean ± SD, n = 3, **P < 0.01. **E** Immunofluorescent section of tumors of tumor-bearing mice treated with free DiD, DiD-L, and CFH/DiD-L at 24 h post-administration. The blue is the nucleus stained by DAPI, the green represents the CAFs labelled with the α-SMA antibody and the red indicates various DiD-labelled formulations

To study the biodistribution of CAFs-targeted liposomes, the stromal-rich xenograft nude mice model was employed in this part. As shown in Fig. 3A, free dye did not distribute at the tumor site but rapidly eliminated from the body. DiD-L presented a visible tumor accumulation at 2–24 h post injection. In comparison, CFH/DiD-L showed an overwhelming fluorescence signal among all the in vivo and ex vivo samples (Fig. 3A, B). Such enhancement of tumoral retention is probably associated with the high affinity between CFH peptide and Tenascin C. As for the tumoral distribution, the fluorescence intensity of tumor excised from CFH/DiD-L-treated mice was 3.24-fold higher than that of DiD-L group (Fig. 3C). After treatment with CFH/DiD-L and DiD-L, the fluorescence signal distributed in both liver and spleen, which is attributed to the liposome-related innate capture by the reticuloendothelial system (Fig. 3D) [28]. According to the fluorescence of various normal tissues at 24 h, the CFH ligand did not influence the biodistribution behaviors (Fig. 3D). Next, the CAFs were marked with green anti-α-SMA antibody, and the intra-tumoral penetration of various formulations labelled with red were further investigated by CLSM. As shown in Fig. 3E, DiD-L primarily accumulated in the outer layer of tumors. In comparison, the signal of CFH/DiD-L significantly overlapped with that of CAFs inside the tumor tissue as indicated by white arrows. These results suggest that the modification of CFH peptide could not only assist the liposome accumulate in the tumor but also distribute around the CAFs, creating a favorable condition for the following regulation of EMT processes.

### Deep penetration in vitro and in vivo

**Fig. 4:**
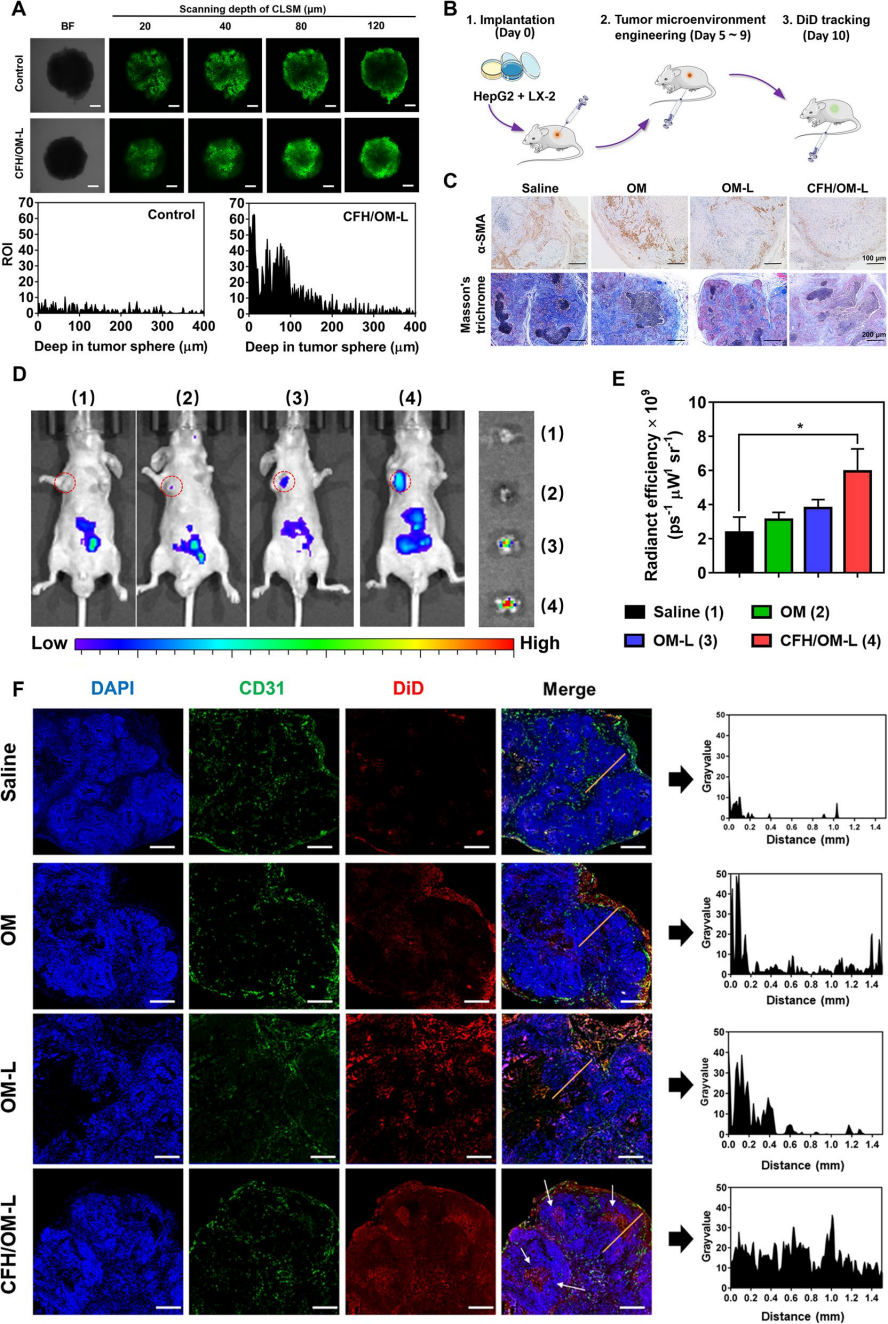
CFH/OM-L promotes deep penetration and intratumor distribution in vitro and vivo. **A** The fluorescence images and quantitative penetration of C6-NPs in 3D tumor spheres treated with CFH/OM-L (100 µM). Scale bar: 200 μm. The intra-3D tumor sphere fluorescence profile is plotted by ImageJ software. **B** Scheme of EMT reversion for tumor deep penetration in vivo. Step 2 represents the administration with various OM formulations and step 3 represents single injection with DiD-NPs. **C** α-SMA and Masson’s trichrome staining of tumor sections in various formulation groups. **D** The NIR images of tumor-bearing mice and ex vivo tumors, DiD-NPs is administrated after 24 h of treatments with the number (1), (2), (3), and (4), which represent the daily administration with saline, OM, OM-L, CFH/OM-L to mice, respectively, and **E** quantitative fluorescence of the ex vivo tumors. Data are represented as mean ± SD, n = 3, *P < 0.05. **F** Immunofluorescence and quantitative intratumoral distribution after treatment with the DiD-NPs at 24 h post the last administration of the OM formulations. The green represents blood vessels stained with CD31 antibody and the red represents the DiD-NPs. The scale bar is 500 μm. The representative region of tumor tissues is indicated by the yellow line (0–1.5 mm) and the corresponding fluorescence intensity profile is plotted through ImageJ software

According to our assumptions, CFH/OM-L can inhibit CAFs activation and collagen expression through reversing EMT process of CAFs, which is theoretically favorable to deep tumor penetration of nanoparticles. To verify CFH/OM-L-resulted deep penetration, the 3D multicellular tumor sphere was employed as the in vitro model. As shown in Fig. 4A, the penetration depth of C6-NPs in CFH/OM-L-pretreated (100 µM) 3D tumor sphere reached 120 μm, which was significantly deeper than that of the control. This is because that CFH/OM-L effectively inhibits the expression of collagen, reducing the diffusion resistance of C6-NPs in the 3D tumor spheres. Notably, the concentration of CFH/OM-L remarkably influenced the penetration of C6-NPs. CFH/OM-L with a low concentration (40 µM) fails to deliver enough OM into the CAFs, and that with a high concentration (200 µM) probably triggers cytotoxicity against tumor sphere, which reduce the overall cellular uptake of 3D tumor spheres (Additional file 1: Fig. S7).

In normal tissues, the distance between the histocyte and blood vessels is about 50–100 μm, whereas tumor cells often reside > 100 μm away from the adjacent blood vessels because of the stroma barriers, hindering the penetration of antitumor drugs [29, 30]. As reported previously, the penetration distance of doxorubicin and paclitaxel was even less than 40– 50 μm after exuding from the tumor blood vessels [31, 32]. As extensively proved, CAFs and collagen notably hinder the delivery of nanoparticles in tumor stroma. In order to investigate the effect of CFH/OM-L on penetration in vivo, CFH/OM-L was administrated into stromal-rich xenograft tumor-bearing nude mice once every day for consecutive 5 days, and the saline, OM and OM-L administration groups were served as controls (Fig. 4B). The expression of α-SMA and collagen of tumor sections in CFH/OM-L-treated mice both significantly decreased compared to that of OM or the saline groups (*P* < 0.05) (Fig. 4C and Additional file 1: Fig. S8). The results suggest that CFH/OM-L can effectively alleviate desmoplasia by inhibiting CAFs activation and collagen deposition in tumor.

Next, we further investigated the intratumoral distribution of second-wave injection of DiD-NPs in vivo. The DiD signal at the tumor of CFH/OM-L-treated mice was 2.5-fold higher than that of the saline group (*P* < 0.05) (Fig. 4D, E), indicating that pre-treated CFH/OM-L was beneficial to DiD-NPs accumulation by alleviating tumor desmoplasia. As shown in Fig. 4F, DiD-NPs were mainly close to the tumor edge (0.1 mm) in the OM-pretreated group. The intratumor penetration of DiD-NPs in OM-L-pretreated group increased up to 0.4 mm, in comparison, a deeper penetration of DiD-NPs (1.5 mm) was observed in CFH/OM-L group. In addition, due to the treatment with CFH/OM-L, the strong red fluorescence was observed in center of the tumor sections as indicated by white arrows, suggesting that CFH/OM-L plays important roles in promoting intratumoral penetration of nanoparticles. It is worth noting that the repeated injections of CFH/OM-L do not result in significant inhibition of tumor growth. Therefore, the anticancer efficacy of combinational CFH/OM-L and IC-ML was studied in the following part.

### Antitumor efficacy toward 3D tumor spheroids

**Fig. 5:**
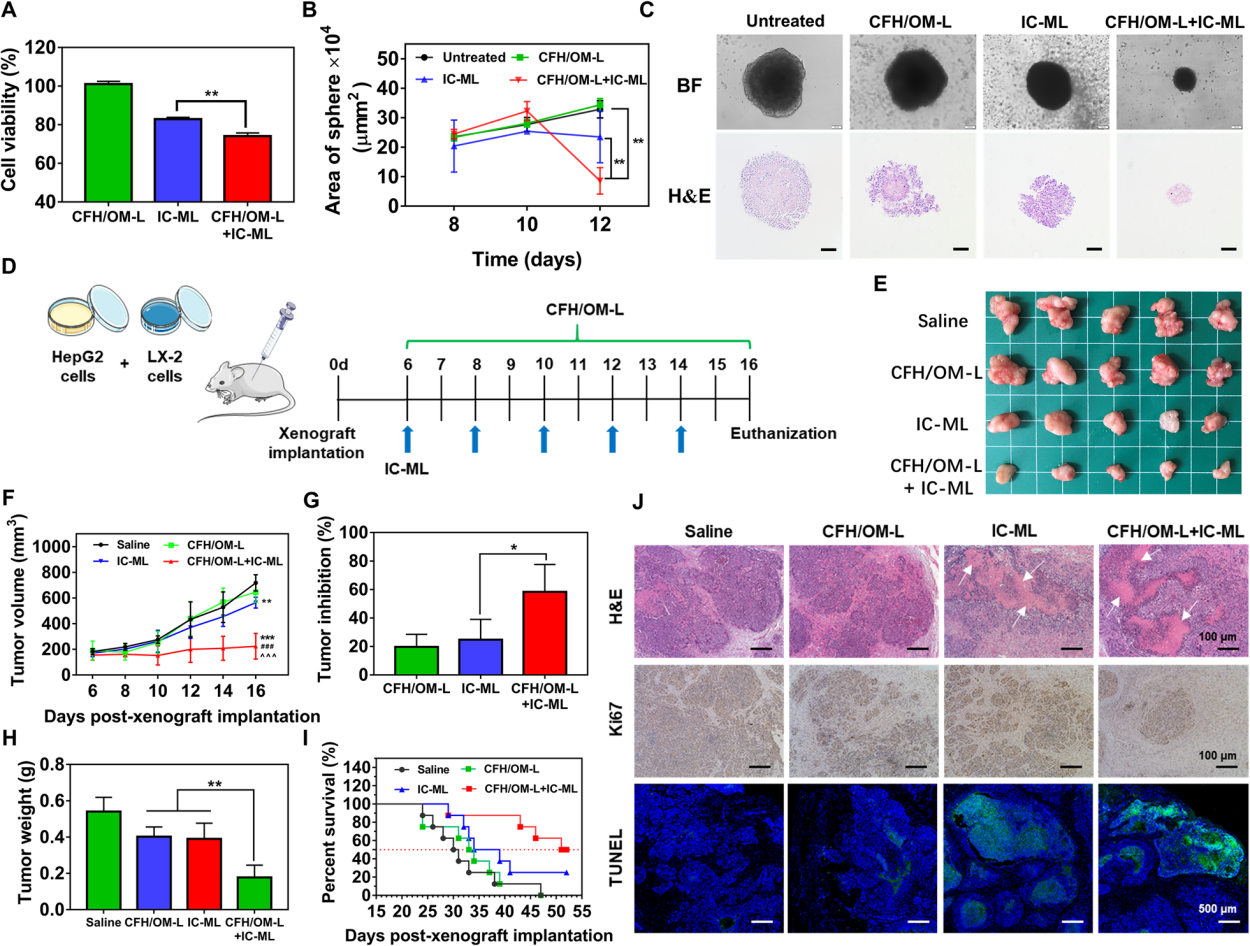
Antitumor efficacy in vitro and vivo. **A** Cell viability of HepG2&LX-2 mixed 3D tumor spheres treated with various formulations. Data are represented as mean ± SD, n = 3, **P < 0.01. **B** Area of 3D tumor spheres after treatments with various formulations. Data are represented as mean ± SD, n = 6, **P < 0.01. **C** Bright field images and H&E staining of the 3D tumor spheres treated with various formulations. Scale bar: 100 μm. **D** Scheme of combinational therapy of CFH/OM-L and IC-ML. **E** Ex vivo tumors of mice at the end of various treatments. **F** Curve of the tumor growth during the treatments. Data are represented as mean ± SD, n = 5. Compared with the saline group, **P < 0.01, ***P < 0.001; compared with IC-ML group, ^###^*P* < 0.001; compared with CFH/OM-L group, ^^^^^*P* < 0.001. **G** Tumor inhibition rate of each group. Data are represented as mean ± SD, n = 5. *P < 0.05. **H** Tumor weight of each group at the end of the treatments. Data are represented as mean ± SD, n = 5. *P < 0.05. **I** Survival curves of tumor-bearing mice during and after the treatments. Data are represented as mean ± SD, n = 8. Compared with the saline group, *P < 0.05. **J** H&E, Ki67, and TUNEL staining of tumor sections in each group

IC-ML is a “small-in-large” lipid complex simultaneously loading icaritin and coix seed oil developed by our group, which is capable of retarding the tumor growth through an anti-angiogenesis strategy [21]. Due to the low administrated dose and limited penetration of large-sized IC-ML, the effectiveness is highly restricted. Here we explored the feasibility of synergism of CFH/OM-L and IC-ML for inhibiting the growth of the 3D tumor spheroids. As shown in Fig. 5A, CFH/OM-L had no obvious cytotoxicity against the 3D multicellular tumor spheroids; however, CFH/OM-L can significantly improve the inhibition of tumor spheroids growth after combined with IC-ML (*P* < 0.01). In addition, the area of the tumor spheroids was recorded in a long-term anti-proliferation study for 12 days. As depicted in Fig. 5B, the tumor sphere area of combined treatment group was significantly lower than that of the untreated group (*P* < 0.01) and IC-ML group (*P* < 0.01) at day 12. The images and H&E-stained sections of tumor spheres were consistent with the above results (Fig. 5C). The structure of 3D tumor spheroids was partially damaged in IC-ML group. Notably, the normal cell nucleuses were barely observed in tumor spheres treated with CFH/OM-L + IC-ML. The above results demonstrate that the combined treatment possesses a synergistic anti-proliferation effect against 3D tumor spheres.

### Antitumor efficacy in vivo

The scheme of the combination treatment with CFH/OM-L and IC-ML was exhibited in Fig. 5D. As shown in Fig. 5E, F, IC-ML slightly restrained the tumor growth probably because of the poor penetration and low dose at the target sites. In comparison, after combinational administration of CFH/OM-L and IC-ML, the size of the stromal-rich xenograft tumor was significantly shrink compared to the other groups. Likewise, the tumor inhibition ratio of CFH/OM-L + IC-ML was beyond 60%, which was obviously higher than that of the two mono treatments (*P* < 0.05) (Fig. 5G). The average tumor weight of mice treated with CFH/OM-L + IC-ML was significantly less than the two mono treatments, and even reduced by 2 times than that of the saline group (Fig. 5H), suggesting the considerable effectiveness of combinational therapy. As for the survival period, CFH/OM-L + IC-ML treatment presented the longest survival time among all the groups (Fig. 5I). As displayed in Fig. 5J, the H&E staining images showed obvious tumor necrosis in CFH/OM-L + IC-ML group. Accordingly, the Ki67-positive area of the tumor sections after treatment with CFH/OM-L + IC-ML was obviously lower than the other groups. TUNEL assay validated that the combinational therapy induced a large number of apoptotic tumor cells, which was consistent with the data about tumor inhibition rate. Overall, combinational CFH/OM-L and IC-ML gained the overwhelming anticancer performance among all the treatments.

### EMT reversion in vivo

**Fig. 6:**
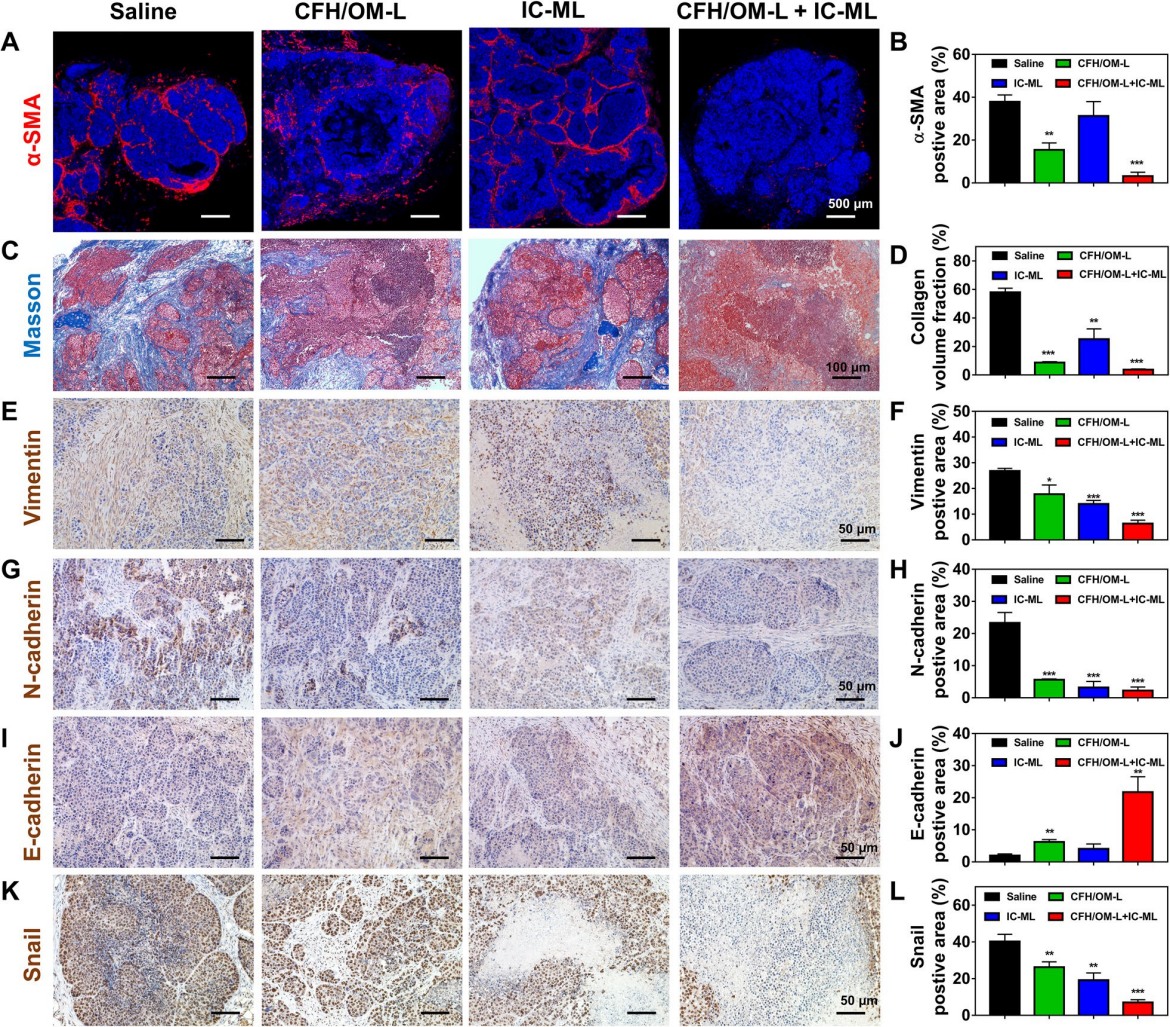
In vivo EMT reversion. **A** Fluorescence images and **B** the corresponding quantification of α-SMA-positive area of tumor sections in various formulation groups. **C** Masson’s trichrome staining images and **D** the corresponding quantification of collagen in various formulation groups. Immunohistochemical staining images and the corresponding quantification of **E**, **F** vimentin, **G, H** N-cadherin, **I**, **J** E-cadherin, and **K**, **L** snail in various formulation groups. All quantitative data are represented as mean ± SD, and quantified by 3 randomly selected observation fields using ImageJ software. Compared with the saline group, *P < 0.05, **P < 0.01, ***P < 0.001

As shown in Fig. 6A–D, the red α-SMA signal and the blue collagen zone in the tumor of CFH/OM-L-treated mice both significantly weakened in comparison to the saline group. Remarkably, the combinational CFH/OM-L and IC-ML further decreased the level of α-SMA and collagen compared to the mono CFH/OM-L group. IC-ML-mediated anti-angiogenesis can alleviate the stubbornness of the TME and thereby hinder the generation of CAFs, which is a potential reason for further reduction of CAFs/collagen in combinational treatment [21]. As the largest population of stromal cells in the TME, CAFs are involved in the secretion of collagen and Th2 cytokines, resulting in the recruitment of immunosuppressive cells, increase of interstitial fluid pressure, and compression of tumor blood vessels, which are response to the obstacles of drug delivery and antitumor therapy [33–37]. According to the previous report, EMT is not only involved in the transformation of CAFs from hepatic stellate cells but also increase invasiveness and metastasis of the tumors [38]. As shown in Fig. 6E–L, compared to the saline-treated mice, the expression of E-cadherin in the tumor sections of CFH/OM-L&IC-ML-treated mice was an increase of ~ 10.98-fold, while the level of N-cadherin, vimentin and snail expression were decrease of ~ 10.31-, ~ 4.22- and ~ 5.71-fold, respectively. The changes in the above-mentioned four markers of EMT process suggest that combinational CFH/OM-L and IC-ML holds promising potential in reversing EMT during the hepatocellular tumorigenesis [39–41].

### TME regulation and characterizations

**Fig. 7:**
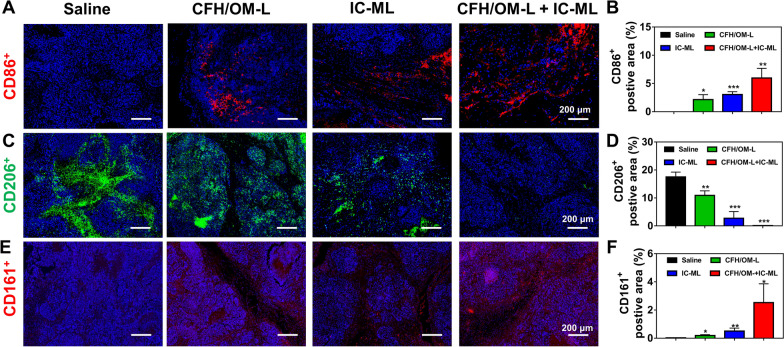
Changes of different immune cell populations in TME. Immunofluorescent staining and quantified area of **A**, **B** CD86^+^-positive M1 TAMs, **C**, **D** CD206^+^-positive M2 TAMs, and **E**,** F** CD161^+^-positive NK cells in tumor tissues of mice treated with various formulations. All quantitative data are represented as mean ± SD, and quantified by 3 randomly selected observation fields using ImageJ software. Compared with the saline group, *P < 0.05, **P < 0.01, ***P < 0.001

CAFs are one of the culprits in the tumor immunosuppressive microenvironment. On the one hand, the desmoplastic TME induced by CAFs hinders the accumulation of immune cells in deep tumor tissue [42]. On the other hand, CAFs interfere with adaptive immune responses by inactivating the functions of dendritic cells and suppressing T cells proliferation & infiltration in the TME [43]. As expected, the combinational treatment with CFH/OM-L and IC-ML not only significantly improved the infiltration CD86^+^ cells but also remarkably suppressed the CD206^+^ population inside the tumors by eliminating collagen barriers constructed by CAFs (Fig. 7A–D), suggesting that such combinational therapy could effectively induce the M1 polarization of TAMs. In addition, the mono administration of IC-ML also able to upregulated the expression of CD86 and downregulated the level of CD206, which might be associated with the crosstalk between anti-angiogenesis and TAMs polarization [21]. Due to this, CFH/OM-L and IC-ML synergistically contribute to the TAMs M1 polarization. The crosstalk between CAFs and immunosuppressive cells would “cold” the TME, further reducing the tumor-killing capacity of immune cells. For example, Mace et al. demonstrated that pancreatic CAFs could repolarize TAMs to the M2 type through producing colony-stimulating factor, IL-6, VEGF, SDF-1 and MCP-1 [44]. Therefore, we infer that the regulatory effect of combination therapy on macrophage M1 polarization might be associated with the crosstalk between CAFs and monocytes/macrophages mediated by cytokines such as TGF-β1 and IL-6.

The NK cells are considered as a positive force for activating the TME; however, the immunosuppressive TME restricts the activity and amount of NK cells, limiting their immune-elimination effectiveness toward the tumor cells [45]. As shown in Fig. 7E, F, all the three treatments significantly increased the amounts of CD161-positive cells of the tumor sections in comparison to the saline group (*P* < 0.05), suggesting that CFH/OM-mediated EMT reversion and IC-ML-mediated anti-angiogenesis collaboratively raised the infiltration of the NK cells [46, 47], which is believed to create a favorable TME for various antitumor treatments. Taken together, the above results demonstrate that the enhancement of the combinational therapy on antitumor efficacy is mainly attributed to the EMT reversion and the resulted TME remodeling.

### Safety evaluation

In order to investigate the safety of combinational CFH/OM-L and IC-ML, the blood routine, liver/spleen index, and H&E staining of main normal tissues were studied. As shown in Additional file 1: Fig. S9, the core indicators of blood routine, such as WBC, RBC, HGB, and PLT, did not present any significant abnormalities after various treatments. As the liver and spleen index, there was no significant changes between the combination therapy and the control (Additional file 1: Fig. S10). According to the H&E-stained sections of the main normal tissues, especially the liver sections, no necrosis or inflammation was observed in different groups (Additional file1: Fig. S11). It again confirms that CFH/OM-L-induced EMT reversion offers a safe approach to reeducate the TME as well as assist other antitumor therapies.

## Conclusions

In summary, we have developed a CAFs-targeted liposomal system (CFH/OM-L) capable of inducing EMT reversion, activating the TME, and promoting intra-tumoral drug delivery of therapeutic nanoparticles (IC-ML). More notably, the combinational CFH/OM-L and IC-ML strengthens the anticancer efficacy via inactivating the production of CAFs, suppressing the expression of collagen, and increasing the infiltration of immunocompetent cells. This study provides a novel CAFs-targeted EMT reversion strategy for TME remolding and adjuvant treatment of hepatocellular carcinoma.

## Materials and methods

### Materials

OM was purchased from Liangwei Biological Technology Co., Ltd (Nanjing, China). Icaritin was obtained from Feiyu Biological Technology Co., Ltd (Nantong, China). Hydrogenated soybean phosphotidylcholine (HSPC) was purchased from AVT Pharmaceutical Tech Co., Ltd (Shanghai, China). Cholesterol was bought from Huixing Co., Ltd (Shanghai, China). 1,2-Distearoyl-sn-glycero-3-phosphatiylethanol-amine-*N*-[methoxy (polyethyleneglycol)-2000] (DSPE-PEG_2000_) was offered by Lipoids GmbH (Ludwigshafen, Germany). CFH peptide (CFHKHKSPALSPVGGG) was synthesized by GL Biochem Co., Ltd (Shanghai, China). DSPE-PEG_2000_-maleimide (DSPE-PEG_2000_-MAL) was provided by Ponsure Biotech, Inc (Shanghai, China). 1,1′-Dioctadecyl-3,3,3′,3′-tetramethylindodicarbocyanine perchlorate (DiD) was obtained from Ruitaibio Co., Ltd (Beijing, China). Coumarin 6 (C6) was purchased from Aladdin Biochemical Technology Co., Ltd (Beijing, China). Cell counting kit-8 (CCK-8) was purchased from Dojindo Laboratories (Tokyo, Japan). The enzyme linked immunosorbent assay (ELISA) kits of collagen I was bought from Elabscience Biotechnology Co., Ltd (Wuhan, China). Tenascin C protein primary antibody was obtained from R&D Systems, Inc. (Minneapolis, MN, USA). The protein primary antibodies of α-SMA, vimentin, N-cadherin, E-cadherin and snail were purchased from Cell Signaling Technology (Danvers, MA, USA). Vimentin, CD86, CD206 and CD161 primary antibodies were offered by Abcam and Biorybt (Cambridge, UK). GAPDH protein primary antibody and AlexaFlour 555/488-labeled secondary antibodies were purchased from Proteintech Group, Inc (IL, USA). Other chemicals were of analytical grade unless otherwise stated.

### Synthesis of DSPE-PEG_2000_-CFH

As reported previously, DSPE-PEG_2000_-MAL and CFH peptide with a molar ratio of 1/2 were stirred in *N*,*N*-dimethylformamide at room temperature [22]. After 4 days of reaction, the raw product was on dialysis (molecular weight cut-off, MWCO: 3000 Da) with running deionized water for 12 h, followed by lyophilization for further use. The chemical structure of DSPE-PEG_2000_-CFH was confirmed by Fourier transform infrared spectroscopy (FT-IR, Tensor 27, Bruker Optik GmbH, Ettlingen, Germany), hydrogen spectrum nuclear magnetic resonance (^1^H NMR, Bruker Optik GmbH, Ettlingen, Germany), and matrix-assisted laser desorption/ionization time-of-flight mass spectrometry (MALDI-TOF MS, Bruker Daltonics, USA).

### Preparation and characterizations CFH/OM-L and IC-ML

CFH/OM-L was prepared by a pH-gradient method as reported previously [16]. Briefly, HSPC/cholesterol/DSPE-PEG_2000_/DSPE-PEG_2000_-CFH with a molar ratio of 38/19/0.5/1 were mixed in 2 mL aliquots of chloroform and obtained a thin lipid film under reduced pressure. The blank liposomes were gained after successive hydration with citric acid solution (150 mM) and ultrasonication. Next, the pH of the liposomes was adjusted to 8.0 via sodium carbonate solution (300 mM), followed by dropping OM solution and incubating in 50 °C to yield CFH/OM-L. Likewise, the OM-L was prepared with a similar method but without DSPE-PEG_2000_-CFH. The FH/OM-L were prepared by a similar method but replacing DSPE-PEG_2000_-CFH with DSPE-PEG_2000_-FH [20]. For fluorescence labelling, C6-loaded liposomes (C6-L and CFH/C6-L) and DiD-loaded liposomes (DiD-L and CFH/DiD-L) were prepared by the aforementioned method but replacing OM with corresponding probes.

The average particle size, zeta potential, polydispersity index (PDI) of the OM-L and CFH/OM-L were measured by dynamic light scattering (DLS, ZetasizerNano 90, Malvern, Worcestershire, UK). The morphology of OM-L and CFH/OM-L were observed with transmission electron microscopy (TEM, HT7700, Hitachi, Tokyo, Japan).

The encapsulation efficiency (EE) of OM-L and CFH/OM-L was determined by the ultrafiltration method as described in the previous report [16]. The unencapsulated drug was separated by the Nanosep tubes (MWCO: 10 kDa, Nanosep, Pall life sciences, USA) at a centrifugation rate of 13,000 rpm. The liposome was dissociated by the 10% Triton X-100 to unload OM. The encapsulation efficiency (EE) was calculated by the following equation, EE (%) = (1 − W_unencapsulated drug_/W_total drug_) × 100%, where W_unencapsulated drug_ and W_total drug_ represent the weight of unencapsulated and the initial feeding OM, respectively.

As reported previously, IC-ML, a small-in-large lipid complex consisting of icaritin and coix seed oil, was prepared by a one-step emulsion & film hydration method [21]. The C6- and DiD-labelled lipid nanoparticles (C6-NPs, DiD-NPs) were also prepared according to our previous report [21]. The surface properties, morphology and other pharmaceutical parameters of IC-ML were characterized with a method reported previously [21].

### Drug release in vitro

Aliquots of CFH/OM-L placed in the dialysis bags (MW: 10 kD) were immersed in various mediums as follows, (1) 0.01 M acetate buffer solution (ABS, pH 5.5); (2) 0.01 M phosphate buffer solution (PBS, pH 6.5), (3) 0.01 M PBS (pH 7.4). Likewise, the release profiles of OM and OM-L were also investigated in 0.01 M PBS (pH 7.4) by the method aforementioned. The accumulative release of OM was determined by HPLC (Waters ACQUITY Arc, Waters Corporation, MA, USA).

### Cell culture

Human hepatic stellate cells (LX-2) and hepatocellular carcinoma cells (HepG2) were cultivated in Dulbecco’s Modified Eagle’s Medium (DMEM) with 10% (v/v) fetal bovine serum, 1% penicillin (100 U/mL), and streptomycin (100 mg/mL) in an incubator with 37 °C and 5% CO_2_.

### Induction of CAFs in vitro

As previous reported [20], LX-2 cells (1 × 10^4^ cells/well) were seed on the circular polylysine-coated glass sheets in 24-well plates for 24 h. The cells were then incubated with DMEM containing 10 ng/mL TGF-β1 for another 24 h. To verify the activation, α-SMA and tenascin C protein were identified by immunofluorescence [48].

### Cellular uptake

Thirty thousand of CAFs were seeded in 24-well plate for 24 h. Next, CAFs were treated with free C6, C6-L, CFH/C6-L and CFH/C6-L + CFH peptide (1 mg/mL) at a C6 concentration of 100 ng/mL for 2 h, followed by rinsing the cells with PBS thrice. Each sample was observed by the fluorescence microscope (VHY-700, Olympus, Tokyo, Japan) immediately. Afterward, the cell suspension was obtained with trypsinization, dispersed in 500 µL of PBS, and then analyzed by flow cytometry (FACSCalibur, BD Biosciences, CA, USA) by counting 10,000 events, successively.

To explore the cellular uptake mechanisms of CFH/C6-L, the TFG-β1-activated CAFs were pretreated with amiloride (133 µg/mL), genistein (54 µg/mL), sucrose (154 mg/mL) at 37 °C, respectively. As well, the cells were also incubated with PBS at 4 °C in advance. After 1 h of preincubation, the CAFs were treated with CFH/C6-L for another 2 h in the presence of above-mentioned inhibitors at 37 °C. The fluorescence intensity of cells was quantified by flow cytometry (FACSCalibur, BD Biosciences, CA, USA).

### EMT reversion in vitro

Fifty thousand of TFG-β1-activated CAFs were seeded into 12-well plate, followed by treating with FBS-free DMEM, OM, OM-L and CFH/OM-L at an OM concentration of 32 µM for 24 h, respectively. α-SMA, vimentin, N-cadherin, E-cadherin and snail proteins in various groups were observed through immunofluorescence staining or western blotting assay according to the standard protocol [25].

The expression of collagen in CAFs was stained by the picrosirius red. Briefly, The CAFs were washed with 500 µL of PBS thrice. Next, 800 µL of 4% paraformaldehyde was added into each well for 15 min, and then washed with 500 µL of PBS twice. Afterward, the cells were stained with 600 µL of picrosirius red staining solution for 30 min, and again rinsed with 1 mL of PBS thrice. After ethanol dehydration and air-dried, the stained cells were observed by the inverted microscope immediately [49]. In addition, the collagen I of CAFs was quantified using by the corresponding ELISA kit (Elabscience, Wuhan, China) according to the standard protocol.

### Penetration in multicellular 3D tumor spheroids

The 3D tumor spheroids of mixed HepG2 and LX-2 cells were constructed according to the previous report [50]. Aliquots of CFH/OM-L at the OM concentration ranging from 40 to 200 µM was incubated with the 3D tumor spheroids for 24 h. Next, C6-NPs at a C6 concentration of 100 ng/mL was added to the 3D tumor spheroids. After 10 h of incubation, 3D tumor spheroids were observed by confocal laser scanning microscope (CLSM, TCS SP8, Leica, Gemmary) to obtain Z-stack images (10 μm interval/scan). The penetration depth of C6-NPs inside the 3D tumor spheroids was quantified by LAS X software.

### Anti-proliferation against multicellular 3D tumor spheroids

3D tumor spheroids were treated with CFH/OM-L, IC-ML and CFH/OM-L + IC-ML for 24 h, respectively. The concentration of IC and OM were set as 8 µM and 40 µM, respectively. After incubation, the tumor spheroids were further stained with the CCK-8 (Dojindo Laboratories, Japan) for 4 h. The absorbance of each well was recorded at 450 nm using a microplate reader (Varioskan Flash; Thermo Fisher Scientific, MA, USA). The cell viability (%) of tumor spheroids was calculated according to advice protocol of CCK-8.

In order to further evaluate the long-term anti-proliferation of various formulations, the 3D tumor spheroids were treated with CFH/OM-L, IC-ML and CFH/OM-L + IC-ML once every two day. The area and morphology of the 3D tumor spheroids were observed using inverted microscope, followed by fixing with 4% paraformaldehyde and staining with hematoxylin/eosin (H&E) according to the protocol.

### Stromal-rich xenograft tumor models

Prior to the experiment, male nude mice (BALB/c, 20 ± 2 g) were acclimated for at least 7 days with free access to food and water in a 12 h light-dark cycle. Two hundred microliters of cell suspension containing HepG2 cells (1 × 10^7^) and LX-2 cells (1 × 10^7^) were subcutaneously injected into the right flank of the nude mice. The stromal-rich xenograft tumor models were considered as success once the tumor size reached 60–80 mm^3^.

### NIR imaging in vivo

Once the xenograft tumor size of the models grew up to 120–150 mm^3^, the stromal-rich mice were randomly assigned to 4 groups (n = 3): saline, free DiD, DiD-L and CFH/DiD-L. The mice were intraperitoneally injected with above-mentioned formulations at a DiD dosage of 200 µg/kg. At 0.5–24 h post administration, the mice were anesthetized by isoflurane and observed with near-infrared (NIR) in vivo imaging system (IVIS Lumina II, Xenogen, USA) to obtain NIR imaging. Next, the mice were euthanized at 24 h post administration. The ex vivo tumor tissues and organs (heart, liver, spleen, lung, kidney) were excised for NIR imaging. The excitation and the emission wavelength were set at 640 and 668 nm, respectively. The fluorescence intensity of tumor tissues and organs were quantified by the region-of-interests tool. After preparation of frozen section, the anti-α-SMA antibody was employed to observe the CAFs by CLSM.

### Characterizations of fibroblast and collagen of tumors

Stromal-rich xenograft tumor-bearing nude mice were randomly-divided into 4 groups (n = 3): saline, OM, OM-L and CFH/OM-L. The mice were intraperitoneally-injected with above-mentioned formulations at an OM dosage of 50 mg/kg, respectively. The administration was performed once every day for 5 days. At the end of the treatment, the mice were sacrificed and the tumors were collected for Masson’s trichrome staining and α-SMA immunohistochemical staining according to the corresponding protocols [51].

### Accumulation and penetration in vivo

Stromal-rich xenograft tumor-bearing nude mice were randomly-divided into 4 groups (n = 3): saline, OM, OM-L and CFH/OM-L. The mice were intraperitoneally-injected with above-mentioned formulations at an OM dosage of 50 mg/kg, respectively. The administration was performed once every day for 5 days. At 24 h post the last OM-based treatment, DiD-NPs were intraperitoneally injected at a DiD dosage of 200 µg/kg. After 24 h, the mice were anesthetized with online isoflurane, and observed by NIR in vivo imaging system (IVIS Lumina II, Xenogen, USA). The penetration of DiD-NPs inside the tumor tissues was quantified by ImageJ software [52].

### Combinational antitumor efficacy in vivo

Fifty-two nude mice bearing stromal-rich xenograft tumors were randomly divided into 4 groups, and the mice were intraperitoneally injected the following formulations: saline, CFH/OM-L, IC-ML and CFH/OM-L + IC-ML. CFH/OM-L (50 mg OM/kg) and IC-ML (1.5 mg IC/kg) were administrated once every day and once every two day, respectively. The tumor size, body weight, and survival time of mice were monitored during the treatment. The long diameter (a) and short diameter (b) of tumors were measured using vernier caliper. The volume of the tumors was calculated according to the formula: V = (a × b^2^)/2. After 16 days of post-xenograft implantation, the tumors of 5 mice randomly-selected from each group were harvested, weighted and prepared the sections. The remaining mice were observed the survival time. The tumor index was calculated as the weight of the tumor to the body weight. The inhibition ratio of tumor was calculated as 1 − (T_test_/T_saline_), where T_test_ and T_saline_ represent the tumor index of the test and saline group, respectively.

H&E staining, TdT-mediated dUTP nick end labeling (TUNEL) staining and immunohistochemistry of Ki67 were performed to evaluate the tumor necrosis, apoptosis, and proliferation, respectively [53]. The Masson staining and immunofluorescence staining of α-SMA were used to characteristic collagen and CAFs of the tumors [54]. The in vivo EMT process of the tumor tissues was verified via immunohistochemical staining of vimentin, N-cadherin, E-cadherin and snail protein by the protocols [55]. The M1 TAMs, M2 TAMs, and NK cells were labelled with anti-CD86, anti-CD206 and anti-CD161 antibodies according to the corresponding protocols, respectively.

### In vivo safety studies

After the treatments, the blood of mice was collected through retro-orbital route. The number of white blood cells (WBC), red blood cells (RBC), hemoglobin (HGB) and platelets (PLT) were determined by automatic hematology analyzer (AU5800, Beckman Coulter, USA). The normal tissues of mice, such as heart, liver, spleen, lung and kidney, were harvested and weighted. The liver/spleen index was calculated as the weight ratio of the liver/spleen tissues to the body. H&E-stained sections of various normal tissues were performed by the corresponding protocol [56].

### Data analysis

All Data presented in this study were shown as a mean ± standard deviation (SD). Statistical tests were performed by SPSS 21.0 statistical software. ANOVA was employed to evaluate the statistical significance. *P < 0.05 and **P < 0.01 represent significant and extremely significant difference, respectively.

## Supplementary Information

**Additional file 1: Figure S1.** Synthesis of DSPE-PEG2000-CFH. **Figure S2.** FT-IR spectrums of (A) FT-IR, (B) DSPE-PEG2000-MAL, and (C) DSPE-PEG2000-CFH. **Figure S3.** 1 H-NMR spectrums of DSPE-PEG2000-MAL and DSPE-PEG2000-CFH. **Figure S4.** MALID-TOF MS spectrums of DSPE-PEG2000-MAL and DSPE-PEG2000-CFH. **Figure S5.** Changes in particle size and zeta potential of CFH/OM-L after incubation with (A) mice plasma for 24 h and (B) PBS under the environment of pH 7.4 for 40 days. (C) Release profile of OM, OM-L and CFH/OM-L in PBS of pH 7.4 for 24 h. Data are represented as mean ± SD, n = 3, ***P < 0.001. **Figure S6.** Particle size distribution and appearance (inserted picture) of IC-ML. **Figure S7.** Fluorescence images (left) and quantitative penetration (right) of C6-NPs in 3D tumor spheres after treated with CFH/OM-L (40 µM, 200 µM). Scale bar: 200 μm. The quantification is calculated with ImageJ software. **Figure S8.** Expression of (A) α-SMA and (B) collagen of tumor sections. Data are represented as mean ± SD. *P < 0.05, **P < 0.01, ***P < 0.001. **Figure S9.** Safety evaluation. (A) WBC, (B) RBC, (C) HGB and (D) PLT after various treatments. Date represents mean ± SD, n = 5. **Figure S10.** (A) Liver and (B) spleen index of mice treated with different formulations. Date represents mean ± SD, n = 5. **Figure S11.** H&E-stained sections of heart, liver, spleen, lung and kidney of mice treated with different formulations. Scale bar: 100 μm.

## Data Availability

All data presented in this paper are included in the main text and the Additional file.

## Abbreviations

CAFs: Cancer-associated fibroblasts
TME: Tumor microenvironment
TAMs: Tumor-associated macrophages
TGF-β: Transforming growth factor-β
CXCL12: C-X-C motif chemokine ligand 12
EMT: Epithelial–mesenchymal transition
OM: Oxymatrine
CFH: Cysteine-end FH peptide
OM-L: OM-loaded liposomes
CFH/OM-L: CFH-decorated OM-loaded liposomes
IC-ML: Lipid complex co-loading icaritin and coix seed oil
HSCs: Hepatic stellate cells
HSPC: Hydrogenated soybean phosphotidylcholine
DSPE-PEG_2000_: 1,2-Distearoyl-sn-glycero-3-phosphatiylethanol-amine-*N*-[methoxy(polyethyleneglycol)-2000]
DSPE-PEG_2000_-MAL: DSPE-PEG_2000_-maleimide
DiD: 1,1′-Dioctadecyl-3,3,3′,3′-tetramethylindodicarbocyanine perchlorate
C6: Coumarin 6
CCK-8: Cell counting kit-8
ELISA: Enzyme linked immunosorbent assay
FT-IR: Fourier transform infrared spectroscopy
^1^H NMR: Hydrogen spectrum nuclear magnetic resonance
MALDI-TOF MS: Matrix-assisted laser desorption/ionization time-of-flight mass spectrometry
C6-L: C6-loaded liposomes
MWCO: Molecular weight cut-off
CFH/C6-L: CFH-decorated C6-loaded liposomes
DiD-L: DiD-loaded liposomes
CFH/DiD-L: CFH-decorated DiD-loaded liposomes
DLS: Dynamic light scattering
TEM: Transmission electron microscopy
C6-NPs: C6-labelled lipid nanoparticles
DiD-NPs: DiD-labelled lipid nanoparticles
PBS: Phosphate buffer solution
H&E: Hematoxylin/eosin
NIR: Near-infrared
TUNEL: TdT-mediated dUTP nick end labeling
WBC: White blood cells
RBC: Red blood cells
HGB: Hemoglobin
PLT: Platelets

## References

[1] Affo S, Yu LX, Schwabe RF (2017). The role of cancer-associated fibroblasts and fibrosis in liver cancer. Annu Rev Pathol.

[2] Truffi M, Mazzucchelli S, Bonizzi A, Sorrentino L, Allevi R, Vanna R (2019). Nano-strategies to target breast cancer-associated fibroblasts: rearranging the tumor microenvironment to achieve antitumor efficacy. Int J Mol Sci.

[3] Sun Q, Zhang B, Hu Q, Qin Y, Xu W, Liu W (2018). The impact of cancer-associated fibroblasts on major hallmarks of pancreatic cancer. Theranostics.

[4] Mishra R, Haldar S, Placencio V, Madhav A, Rohena-Rivera K, Agarwal P (2018). Stromal epigenetic alterations drive metabolic and neuroendocrine prostate cancer reprogramming. J Clin Invest.

[5] Zhang W, Bouchard G, Yu A, Shafiq M, Jamali M, Shrager JB (2018). GFPT2-expressing cancer-associated fibroblasts mediate metabolic reprogramming in human lung adenocarcinoma. Cancer Res.

[6] Miao L, Newby JM, Lin CM, Zhang L, Xu F, Kim WY (2016). The binding site barrier elicited by tumor-associated fibroblasts interferes disposition of nanoparticles in stroma-vessel type tumors. ACS Nano.

[7] Balkwill FR, Capasso M, Hagemann T (2012). The tumor microenvironment at a glance. J Cell Sci.

[8] Rhim AD, Oberstein PE, Thomas DH, Mirek ET, Palermo CF, Sastra SA (2014). Stromal elements act to restrain, rather than support, pancreatic ductal adenocarcinoma. Cancer Cell.

[9] Özdemir BC, Pentcheva-Hoang T, Carstens JL, Zheng X, Wu CC, Simpson TR (2014). Depletion of carcinoma-associated fibroblasts and fibrosis induces immunosuppression and accelerates pancreas cancer with reduced survival. Cancer Cell.

[10] Lang J, Zhao X, Qi Y, Zhang Y, Han X, Ding Y (2019). Reshaping prostate tumor microenvironment to suppress metastasis via cancer-associated fibroblast inactivation with peptide-assembly-based nanosystem. ACS Nano.

[11] Baglieri J, Brenner DA, Kisseleva T (2019). The role of fibrosis and liver-associated fibroblasts in the pathogenesis of hepatocellular carcinoma. Int J Mol Sci.

[12] Li J, Hu L, Zhou T, Gong X, Jiang R, Li H (2019). Taxifolin inhibits breast cancer cells proliferation, migration and invasion by promoting mesenchymal to epithelial transition via β-catenin signaling. Life Sci.

[13] Shetti D, Zhang B, Fan C, Mo C, Lee BH, Wei K (2019). Low dose of paclitaxel combined with XAV939 attenuates metastasis, angiogenesis and growth in breast cancer by suppressing Wnt signaling. Cells.

[14] Zhao HW, Zhang ZF, Chai X, Li GQ, Cui HR, Wang HB (2016). Oxymatrine attenuates CCl4-induced hepatic fibrosis via modulation of TLR4-dependent inflammatory and TGF-β1 signaling pathways. Int Immunopharmacol.

[15] Cao J, Sun J, Wang X, Li X, Deng Y (2009). N-trimethyl chitosan-coated multivesicular liposomes for oxymatrine oral delivery. Drug Dev Ind Pharm.

[16] Liu M, Jin S, Yan H, Du S (2017). Effect of oxymatrine HSPC liposomes on improving bioavailability, liver target distribution and hepatoprotective activity of oxymatrine. Eur J Pharm Sci.

[17] Kalluri R (2016). The biology and function of fibroblasts in cancer. Nat Rev Cancer.

[18] Chiquet-Ehrismann R, Tucker RP (2011). Tenascins and the importance of adhesion modulation. Cold Spring Harb Perspect Biol.

[19] Kim MY, Kim OR, Choi YS, Lee H, Park K, Lee CT (2012). Selection and characterization of tenascin C targeting peptide. Mol Cells.

[20] Chen B, Wang Z, Sun J, Song Q, He B, Zhang H (2016). A tenascin C targeted nanoliposome with navitoclax for specifically eradicating of cancer-associated fibroblasts. Nanomedicine.

[21] Guo J, Zeng H, Liu Y, Shi X, Liu Y, Liu C (2021). Multicomponent thermosensitive lipid complexes enhance desmoplastic tumor therapy through boosting anti-angiogenesis and synergistic strategy. Int J Pharm.

[22] Yang F-Y, Teng M-C, Lu M, Liang H-F, Lee Y-R, Yen C-C (2012). Treating glioblastoma multiforme with selective high-dose liposomal doxorubicin chemotherapy induced by repeated focused ultrasound. Int J Nanomed.

[23] Chen B, Dai W, Mei D, Liu T, Li S, He B (2016). Comprehensively priming the tumor microenvironment by cancer-associated fibroblast-targeted liposomes for combined therapy with cancer cell-targeted chemotherapeutic drug delivery system. J Control Release.

[24] Li W, Little N, Park J, Foster CA, Chen J, Lu J (2021). Tumor-associated fibroblast-targeting nanoparticles for enhancing solid tumor therapy: progress and challenges. Mol Pharm.

[25] Chen Y, Chen L, Zhang JY, Chen ZY, Liu TT, Zhang YY (2019). Oxymatrine reverses epithelial–mesenchymal transition in breast cancer cells by depressing α(V)β(3) integrin/FAK/PI3K/Akt signaling activation. Onco Targets Ther.

[26] Liang L, Wu J, Luo J, Wang L, Chen ZX, Han CL (2020). Oxymatrine reverses 5-fluorouracil resistance by inhibition of colon cancer cell epithelial–mesenchymal transition and NF-κB signaling in vitro. Oncol Lett.

[27] Izdebska M, Zielińska W, Hałas-Wiśniewska M, Mikołajczyk K, Grzanka A (2019). The cytotoxic effect of oxymatrine on basic cellular processes of A549 non-small lung cancer cells. Acta Histochem.

[28] Crommelin DJA, van Hoogevest P, Storm G (2020). The role of liposomes in clinical nanomedicine development. What now? Now what?. J Control Release.

[29] Khawar IA, Kim JH, Kuh HJ (2015). Improving drug delivery to solid tumors: priming the tumor microenvironment. J Control Release.

[30] Minchinton AI, Tannock IF (2006). Drug penetration in solid tumours. Nat Rev Cancer.

[31] Kyle AH, Huxham LA, Yeoman DM, Minchinton AI (2007). Limited tissue penetration of taxanes: a mechanism for resistance in solid tumors. Clin Cancer Res.

[32] Primeau AJ, Rendon A, Hedley D, Lilge L, Tannock IF (2005). The distribution of the anticancer drug doxorubicin in relation to blood vessels in solid tumors. Clin Cancer Res.

[33] Padera TP, Stoll BR, Tooredman JB, Capen D, di Tomaso E, Jain RK (2004). Pathology: cancer cells compress intratumour vessels. Nature.

[34] Stylianopoulos T, Martin JD, Chauhan VP, Jain SR, Diop-Frimpong B, Bardeesy N (2012). Causes, consequences, and remedies for growth-induced solid stress in murine and human tumors. Proc Natl Acad Sci USA.

[35] Miao L, Lin CM, Huang L (2015). Stromal barriers and strategies for the delivery of nanomedicine to desmoplastic tumors. J Control Release.

[36] Chen X, Song E (2019). Turning foes to friends: targeting cancer-associated fibroblasts. Nat Rev Drug Discov.

[37] Heldin CH, Rubin K, Pietras K, Ostman A (2004). High interstitial fluid pressure—an obstacle in cancer therapy. Nat Rev Cancer.

[38] Ribatti D, Tamma R, Annese T (2020). Epithelial–mesenchymal transition in cancer: a historical overview. Transl Oncol.

[39] Labernadie A, Kato T, Brugués A, Serra-Picamal X, Derzsi S, Arwert E (2017). A mechanically active heterotypic E-cadherin/N-cadherin adhesion enables fibroblasts to drive cancer cell invasion. Nat Cell Biol.

[40] Li X, Chen H, Liu Z, Ye Z, Gou S, Wang C (2018). Overexpression of MIST1 reverses the epithelial–mesenchymal transition and reduces the tumorigenicity of pancreatic cancer cells via the Snail/E-cadherin pathway. Cancer Lett.

[41] Pastushenko I, Brisebarre A, Sifrim A, Fioramonti M, Revenco T, Boumahdi S (2018). Identification of the tumour transition states occurring during EMT. Nature.

[42] Salmon H, Franciszkiewicz K, Damotte D, Dieu-Nosjean M-C, Validire P, Trautmann A (2012). Matrix architecture defines the preferential localization and migration of T cells into the stroma of human lung tumors. J Clin Invest.

[43] Nagarsheth N, Wicha MS, Zou W (2017). Chemokines in the cancer microenvironment and their relevance in cancer immunotherapy. Nat Rev Immunol.

[44] Mace TA, Ameen Z, Collins A, Wojcik S, Mair M, Young GS (2013). Pancreatic cancer-associated stellate cells promote differentiation of myeloid-derived suppressor cells in a STAT3-dependent manner. Cancer Res.

[45] Terrén I, Orrantia A, Vitallé J, Zenarruzabeitia O, Borrego F (2019). NK cell metabolism and tumor microenvironment. Front Immunol.

[46] Hydes T, Abuhilal M, Armstrong T, Primrose J, Takhar A, Khakoo S (2015). Natural killer cell maturation markers in the human liver and expansion of an NKG2C+ KIR+ population. Lancet.

[47] Iiai T, Watanabe H, Suda T, Okamoto H, Abo T, Hatakeyama K (2002). CD161+ T (NT) cells exist predominantly in human intestinal epithelium as well as in liver. Clin Exp Immunol.

[48] Chen Y, Qu D, Fu R, Guo M, Qin Y, Guo J (2018). A Tf-modified tripterine-loaded coix seed oil microemulsion enhances anti-cervical cancer treatment. Int J Nanomed.

[49] Zhao L, Xu Y, Tao L, Yang Y, Shen X, Li L (2018). Oxymatrine inhibits transforming growth factor β1 (TGF-β1)-induced cardiac fibroblast-to-myofibroblast transformation (FMT) by mediating the notch signaling pathway in vitro. Med Sci Monit.

[50] Qu D, Wang L, Qin Y, Guo M, Guo J, Huang M (2018). Non-triggered sequential-release liposomes enhance anti-breast cancer efficacy of STS and celastrol-based microemulsion. Biomater Sci.

[51] Miao L, Liu Q, Lin CM, Luo C, Wang Y, Liu L (2017). Targeting tumor-associated fibroblasts for therapeutic delivery in desmoplastic tumors. Cancer Res.

[52] Xu H, Hu M, Liu M, An S, Guan K, Wang M (2020). Nano-puerarin regulates tumor microenvironment and facilitates chemo- and immunotherapy in murine triple negative breast cancer model. Biomaterials.

[53] Yang Q, Li L, Sun W, Zhou Z, Huang Y (2016). Dual stimuli-responsive hybrid polymeric nanoparticles self-assembled from POSS-based starlike copolymer-drug conjugates for efficient intracellular delivery of hydrophobic drugs. ACS Appl Mater Interfaces.

[54] Ji T, Ding Y, Zhao Y, Wang J, Qin H, Liu X (2015). Peptide assembly integration of fibroblast-targeting and cell-penetration features for enhanced antitumor drug delivery. Adv Mater.

[55] Wei C, Yang C, Wang S, Shi D, Zhang C, Lin X (2019). Crosstalk between cancer cells and tumor associated macrophages is required for mesenchymal circulating tumor cell-mediated colorectal cancer metastasis. Mol Cancer.

[56] Qin Y, Liu T, Guo M, Liu Y, Liu C, Chen Y (2020). Mild-heat-inducible sequentially released liposomal complex remodels the tumor microenvironment and reinforces anti-breast-cancer therapy. Biomater Sci.

