# Supplementary material for: A CFH peptide-decorated liposomal oxymatrine inactivates cancer-associated fibroblasts of hepatocellular carcinoma through epithelial–mesenchymal transition reversion
Source: J Nanobiotechnology. 2022 Mar 5;20:114. doi: 10.1186/s12951-022-01311-1 (PMC8898522; doi:10.1186/s12951-022-01311-1)
Supplement: Supplementary file 1 — Additional file 1: Figure S1. Synthesis of DSPE-PEG2000-CFH. Figure S2. FT-IR spectrums of (A) FT-IR, (B) DSPE-PEG2000-MAL, and (C) DSPE-PEG2000-CFH. Figure S3. 1 H-NMR spectrums of DSPE-PEG2000-MAL and DSPE-PEG2000-CFH. Figure S4. MALID-TOF MS spectrums of DSPE-PEG2000-MAL and DSPE-PEG2000-CFH. Figure S5. Changes in particle size and zeta potential of CFH/OM-L after incubation with (A) mice plasma for 24 h and (B) PBS under the environment of pH 7.4 for 40 days. (C) Release profile of OM, OM-L and CFH/OM-L in PBS of pH 7.4 for 24 h. Data are represented as mean ± SD, n = 3, ***P < 0.001. Figure S6. Particle size distribution and appearance (inserted picture) of IC-ML. Figure S7. Fluorescence images (left) and quantitative penetration (right) of C6-NPs in 3D tumor spheres after treated with CFH/OM-L (40 µM, 200 µM). Scale bar: 200 μm. The quantification is calculated with ImageJ software. Figure S8. Expression of (A) α-SMA and (B) collagen of tumor sections. Data are represented as mean ± SD. *P < 0.05, **P < 0.01, ***P < 0.001. Figure S9. Safety evaluation. (A) WBC, (B) RBC, (C) HGB and (D) PLT after various treatments. Date represents mean ± SD, n = 5. Figure S10. (A) Liver and (B) spleen index of mice treated with different formulations. Date represents mean ± SD, n = 5. Figure S11. H&E-stained sections of heart, liver, spleen, lung and kidney of mice treated with different formulations. Scale bar: 100 μm. [file 12951_2022_1311_MOESM1_ESM.docx]

**Additional file 1**

**A CFH peptide-decorated liposomal oxymatrine** **inactivates cancer-associated fibroblasts of hepatocellular** **carcinoma through epithelial-mesenchymal transition reversion**

Jian Guo^1,2,3^, Huating Zeng^1^, Xinmeng Shi^1^, Tao Han^1^, Yimin Liu^1^, Yuping Liu^1,2^, Congyan Liu^1,2^, Ding Qu ^1,2,*^, Yan Chen^1,2,*^

^1^Affiliated Hospital of Integrated Traditional Chinese and Western Medicine, Nanjing University of Chinese Medicine, Nanjing 210028, China

^2^Jiangsu Province Academy of Traditional Chinese Medicine, Nanjing 210028, China

^3^Anhui Province Key Laboratory of Pharmaceutical Preparation Technology and Application, College of Pharmacy, Anhui University of Chinese Medicine, Hefei 230012, China

*Corresponding author:

Ding Qu, Ph.D., Professor, Jiangsu Province Academy of Chinese Medicine, 100 Shizi Road, Nanjing 210028, China. E-mail: quding1985@hotmail.com

Yan Chen, Ph.D., Professor, Jiangsu Province Academy of Chinese Medicine, 100 Shizi Road, Nanjing 210028, China. E-mail: ychen202@hotmail.com.


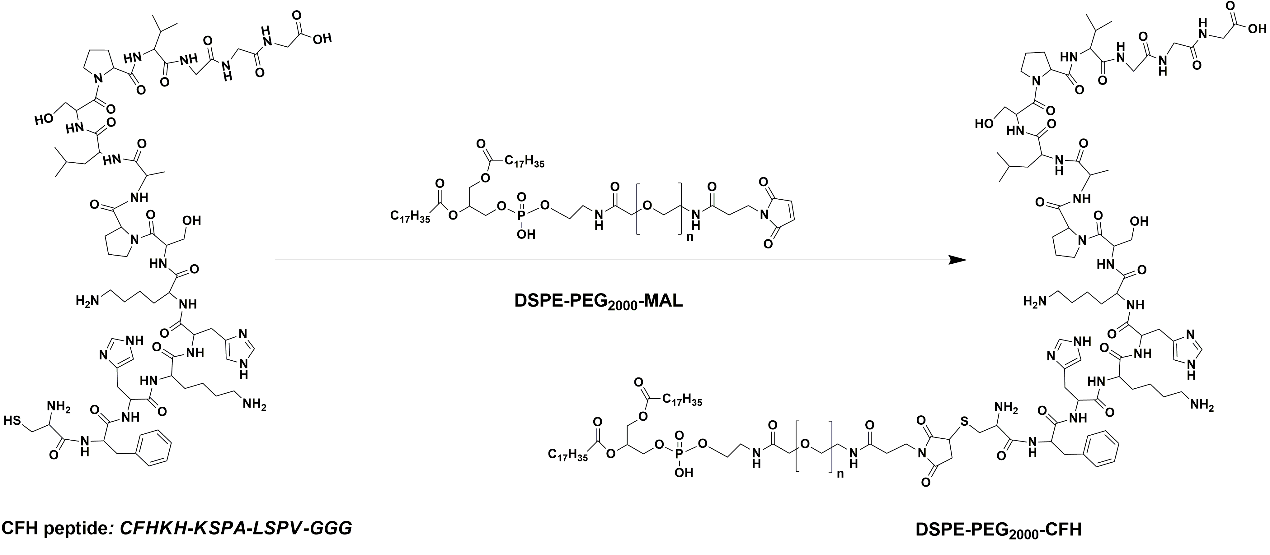


**Fig. S1** Synthesis of DSPE-PEG_2000_-CFH


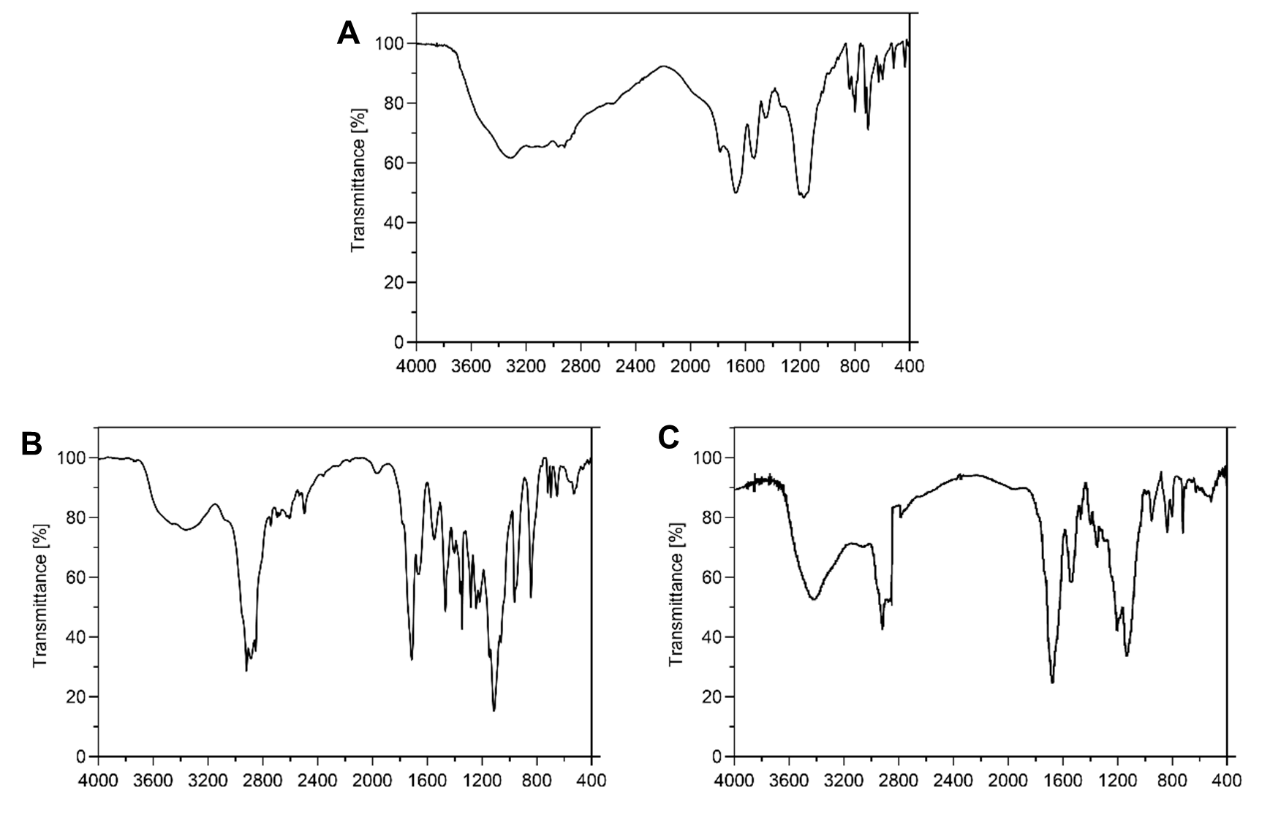


**Fig. S2** FT-IR spectrums of (**A**) FT-IR, (**B**) DSPE-PEG_2000_-MAL, and (**C**) DSPE-PEG_2000_-CFH


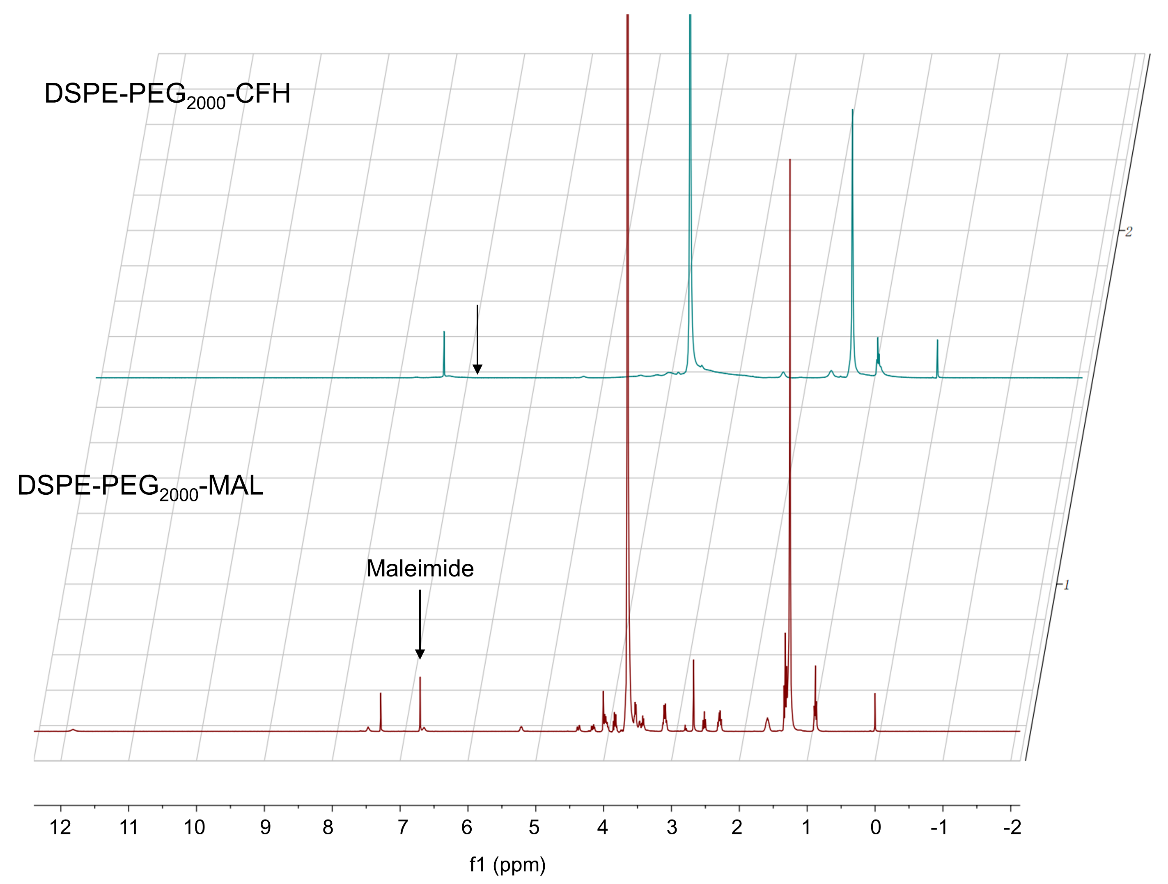


**Fig. S3** ^1^H-NMR spectrums of DSPE-PEG_2000_-MAL and DSPE-PEG_2000_-CFH


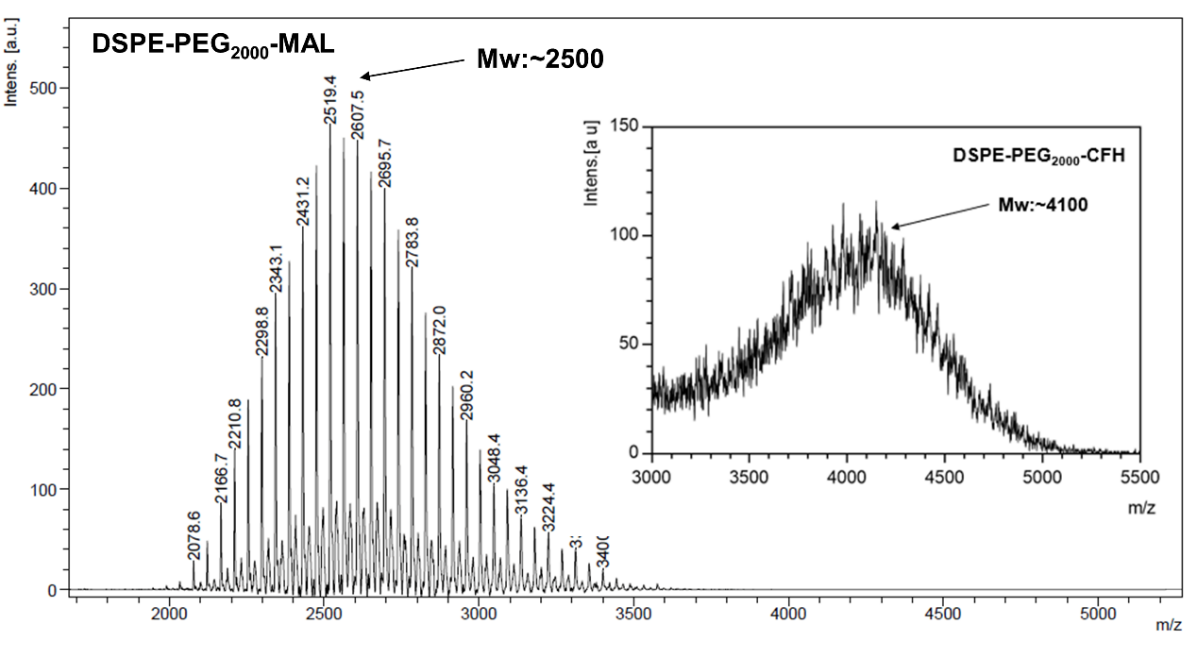


**Fig. S4** MALID-TOF MS spectrums of DSPE-PEG_2000_-MAL and DSPE-PEG_2000_-CFH.


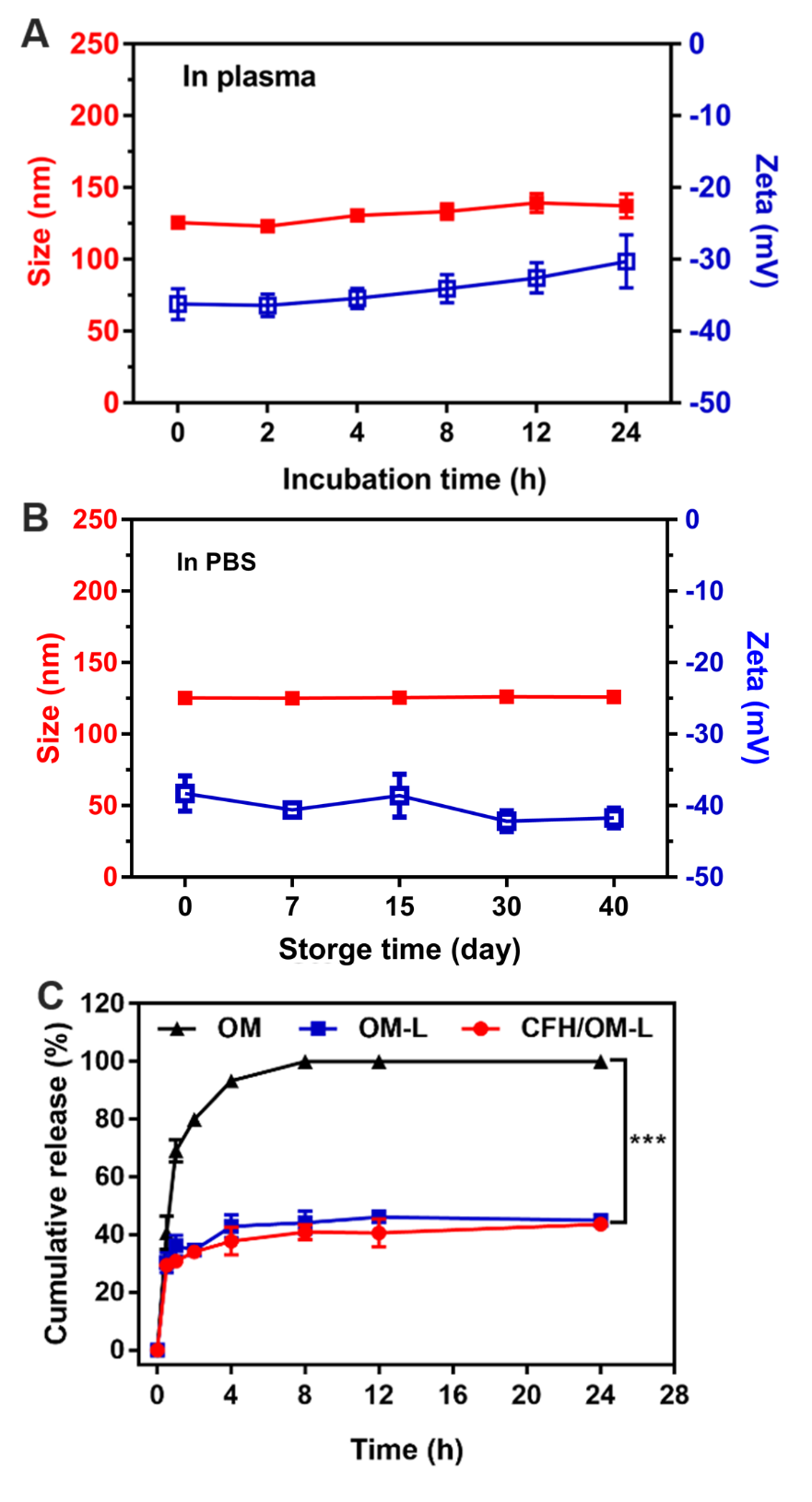


**Fig. S5** Changes in particle size and zeta potential of CFH/OM-L after incubation with (**A**) mice plasma for 24 h and (**B**) PBS under the environment of pH 7.4 for 40 days. (**C**) Release profile of OM, OM-L and CFH/OM-L in PBS of pH 7.4 for 24 h. Data are represented as mean ± SD, n = 3, ****P* < 0.001.


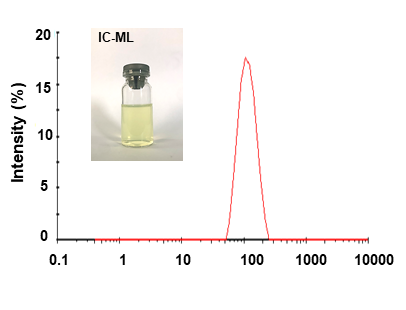


**Fig. S6** Particle size distribution and appearance (inserted picture) of IC-ML.


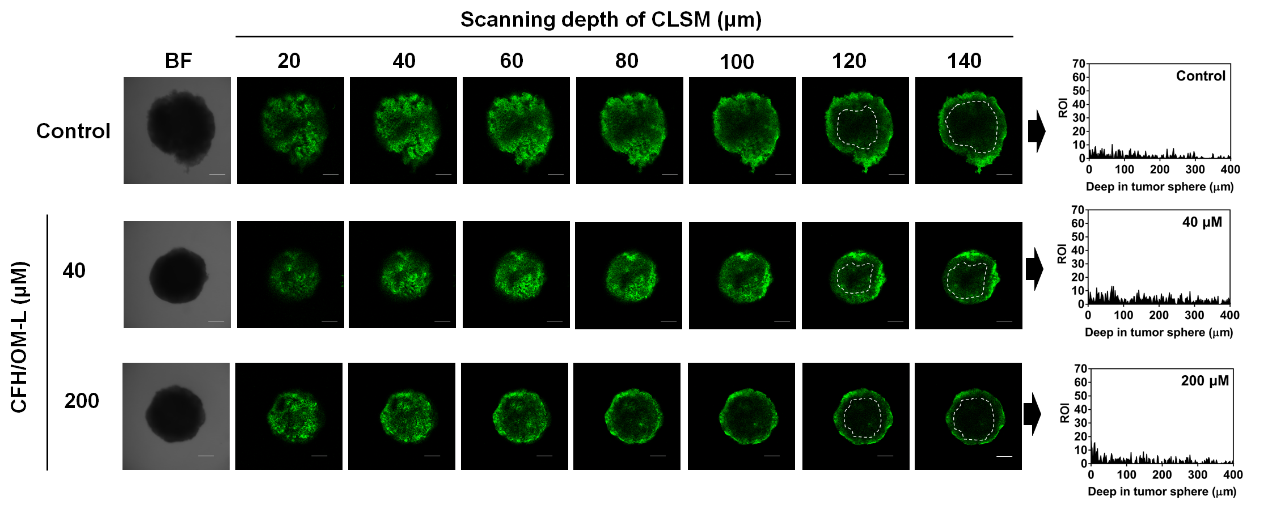


**Fig. S7** Fluorescence images (left) and quantitative penetration (right) of C6-NPs in 3D tumor spheres after treated with CFH/OM-L (40μM, 200μM). Scale bar: 200 μm. The quantification is calculated with ImageJ software.


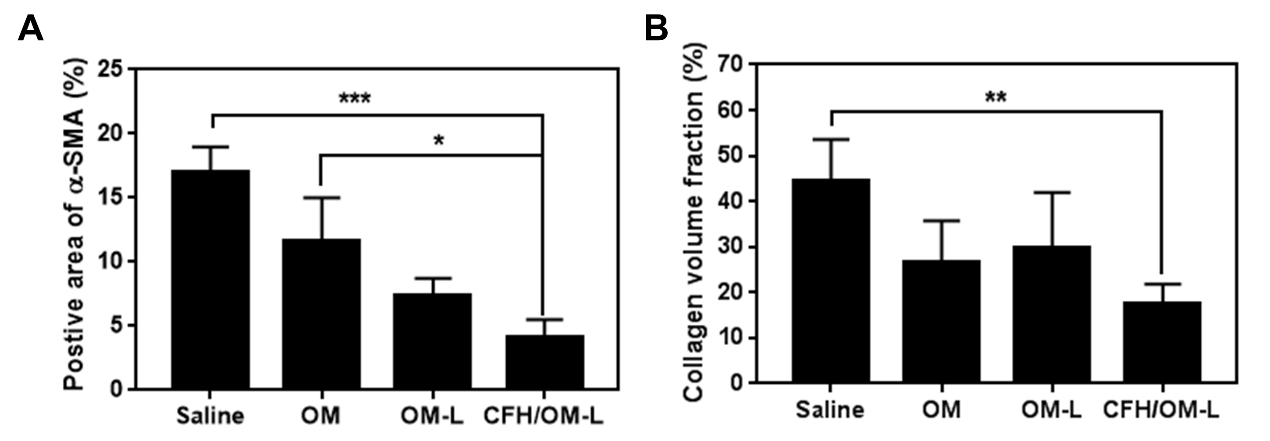


**Fig. S8** Expression of (**A**) α-SMA and (**B**) collagen of tumor sections. Data are represented as mean ± SD. ^*^*P* < 0.05, ^**^*P* < 0.01, *^***^P* < 0.001.


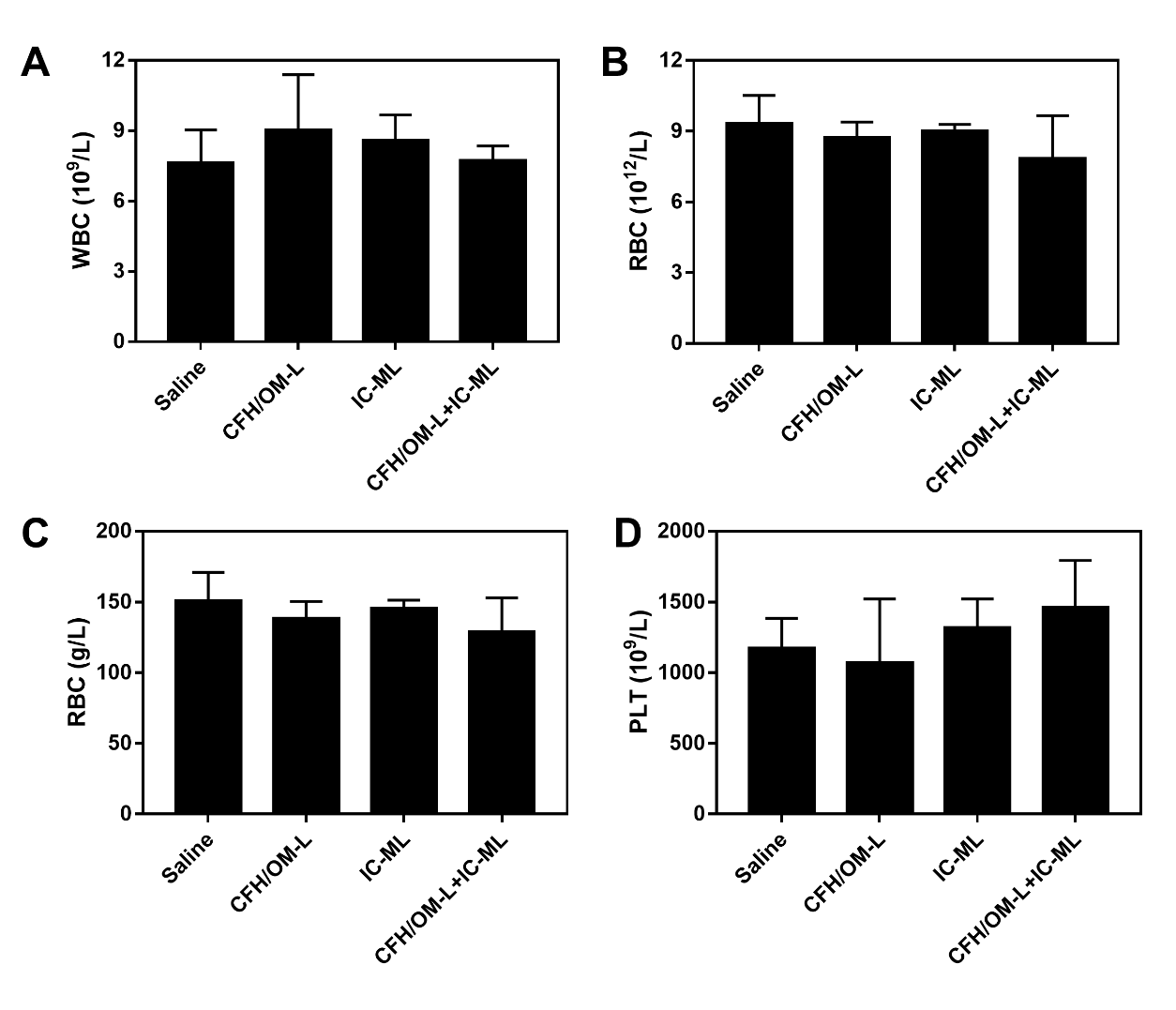


**Fig. S9** Safety evaluation. (**A**) WBC, (**B**) RBC, (**C**) HGB and (**D**) PLT after various treatments. Date represents mean ± SD, n = 5.


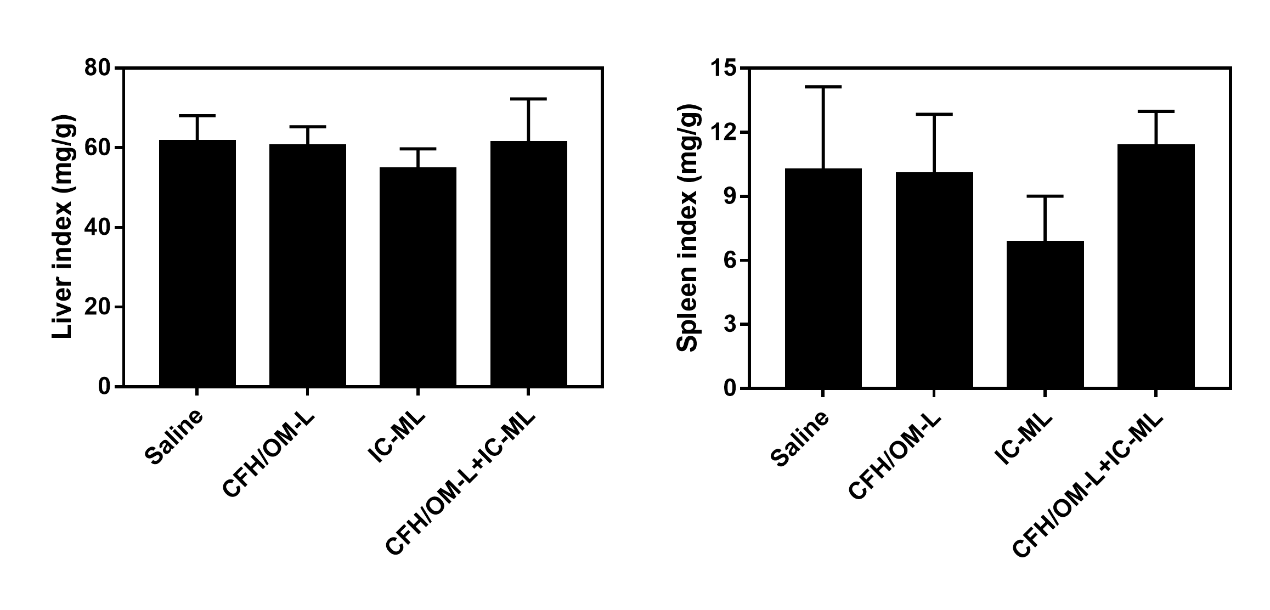


**Fig. S10** (**A**) Liver and (**B**) spleen index of mice treated with different formulations. Date represents mean ± SD, n = 5.


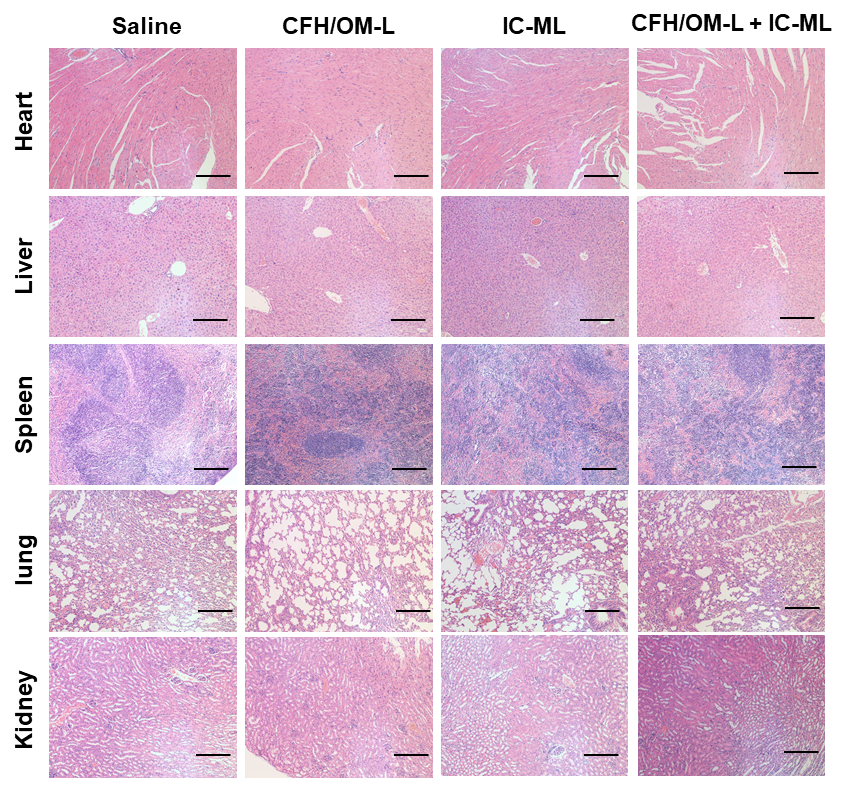


**Fig. S11** H&E-stained sections of heart, liver, spleen, lung and kidney of mice treated with different formulations. Scale bar: 100 μm.
